# Peptide Model of the Mutant Proinsulin Syndrome. II. Nascent Structure and Biological Implications

**DOI:** 10.3389/fendo.2022.821091

**Published:** 2022-03-01

**Authors:** Yanwu Yang, Michael D. Glidden, Balamurugan Dhayalan, Alexander N. Zaykov, Yen-Shan Chen, Nalinda P. Wickramasinghe, Richard D. DiMarchi, Michael A. Weiss

**Affiliations:** ^1^Department of Biochemistry and Molecular Biology, Indiana University School of Medicine, Indianapolis, IN, United States; ^2^Department of Biochemistry, Case Western Reserve University School of Medicine, Cleveland, OH, United States; ^3^Department of Physiology & Biophysics, Case Western Reserve University School of Medicine, Cleveland, OH, United States; ^4^Department of Medicine, Case Western Reserve University School of Medicine, Cleveland, OH, United States; ^5^Department of Chemistry, Indiana University, Bloomington, IN, United States

**Keywords:** monogenic diabetes, peptide chemistry, protein folding, folding nucleus, oxidative folding intermediate, NMR spectroscopy

## Abstract

Toxic misfolding of proinsulin variants in β-cells defines a monogenic diabetes syndrome, designated *mutant INS-gene induced diabetes of the young* (MIDY). In our first study (previous article in this issue), we described a one-disulfide peptide model of a proinsulin folding intermediate and its use to study such variants. The mutations (Leu^B15^→Pro, Leu^A16^→Pro, and Phe^B24^→Ser) probe residues conserved among vertebrate insulins. In this companion study, we describe ^1^H and ^1^H-^13^C NMR studies of the peptides; key NMR resonance assignments were verified by synthetic ^13^C-labeling. Parent spectra retain nativelike features in the neighborhood of the single disulfide bridge (cystine B19-A20), including secondary NMR chemical shifts and nonlocal nuclear Overhauser effects. This partial fold engages wild-type side chains Leu^B15^, Leu^A16^ and Phe^B24^ at the nexus of nativelike α-helices α_1_ and α_3_ (as defined in native proinsulin) and flanking β-strand (residues B24-B26). The variant peptides exhibit successive structural perturbations in order: parent (most organized) > Ser^B24^ >> Pro^A16^ > Pro^B15^ (least organized). The same order pertains to (a) overall α-helix content as probed by circular dichroism, (b) synthetic yields of corresponding three-disulfide insulin analogs, and (c) ER stress induced in cell culture by corresponding mutant proinsulins. These findings suggest that this and related peptide models will provide a general platform for classification of MIDY mutations based on molecular mechanisms by which nascent disulfide pairing is impaired. We propose that the syndrome’s variable phenotypic spectrum—onsets ranging from the neonatal period to later in childhood or adolescence—reflects structural features of respective folding intermediates.

## Introduction

The mutant proinsulin syndrome (MPS) is a prototypical disease of toxic protein misfolding. Unlike toxic *extracellular* aggregation as observed among neurodegenerative diseases (1) and diverse amyloid disorders (2), in the MPS a dominant mutation impairs proinsulin folding efficiency in a critical *intracellular* organelle: the endoplasmic reticulum (ER). Impaired foldability of the variant protein of pancreatic β-cells leads to aberrant aggregation and in turn induces chronic ER stress (3–5). Although the unfolded protein response (UPR) evolved as an adaptive pathway [broadly conserved among eukaryotic cells (6, 7)], its chronic activation in β-cells impairs glucose-stimulated insulin secretion and β-cell viability [for review, see (8, 9)]. Also designated *mutant INS-gene induced diabetes of the young* (MIDY) (4), MPS encompasses a range of patient phenotypes, representing subtypes of *permanent neonatal diabetes mellitus* (PNDM) to *maturity-onset diabetes of the young* (MODY) (10–13).

Among monogenic endocrine syndromes in general [such as complete or partial androgen insensitivity (14)], a given mutation may be associated with a range of phenotypes, even in the same kindred (15). A given mutation in the androgen receptor, for example, may be associated with male development, somatic female development with Mullerian regression, or ambiguous genitalia (16). The complexity of genotype-phenotype relationships (GPR) in such syndromes presumably reflects the influence of modifier genes in multigenic regulatory pathways (17). Modifier genes have also been inferred in the genetics of Type 1 diabetes mellitus (DM). GPRs in MPS may be more straightforward: the extent of β-cell dysfunction and velocity of β-cell loss (together determining age of diabetes onset in a particular patient) may reflect mutation-specific molecular properties, i.e., whether a given amino-acid substitution is associated with a severe (PNDM) or mild (MODY) perturbation to foldability. In our initial study [preceding article in this issue (18)] we designed a 49-residue peptide model of an early on-pathway proinsulin folding intermediate and its application to three representative MIDY mutations. This single-chain model contains only one disulfide bridge and is thus designated *1SS*. This bridge (cystine B19-A20) is the first to accumulate among populated partial folds in the *in vitro* folding pathway of proinsulin and homologous factors (19, 20). The present study provides a detailed two-dimensional NMR study of the parent 1SS peptide and representative MIDY-related variants.

**Figure 1:**
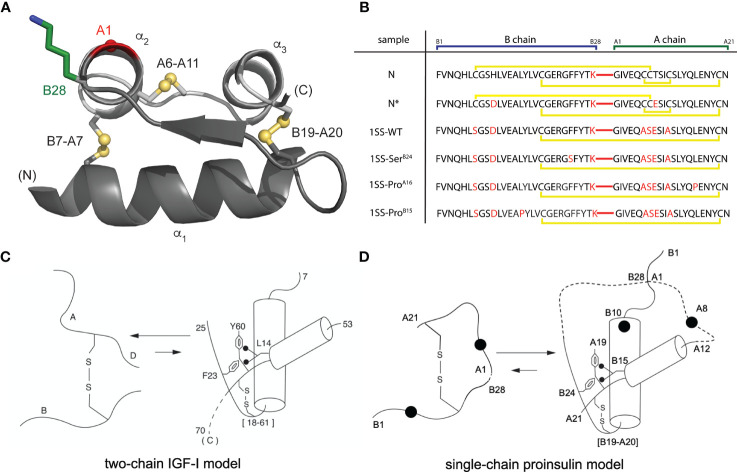
Single-chain DesDi as a proinsulin model. **(A)** Cartoon model of single-chain DesDi molecule that connects A- and B- chains through a linkage between Lys^B28^ and Gly^A1^ (PDB entry 7JP3). Color legend: B chain, dark gray; A chain, light gray; Lys^B28^ side chain, green sticks; Gly^A1^-C_α_, red sphere; sulfur atoms in disulfide bonds are shown as spheres. All the spheres are set to one third Van der walls radii. N- and C- terminals are indicated. Insulin’s three characteristic helices are labeled as α_1_, α_2_, and α_3_. **(B)** DesDi protein sequences. Sample names at left report the presence of either one ([B19-A20]) or three (N and N*) disulfide bonds; yellow lines show disulfide linkages. A red line connecting the C-terminal B-domain and N-terminal A-domain sequences signifies the presence of a peptide bond between residues B28 and A1. All [B19-A20]-1SS samples and N* have additional mutations Glu^A8^ and Asp^B10^ to enhance solubility at neutral pH and the high protein concentrations needed for NMR spectroscopy. **(C)** General folding nucleus of insulin-related superfamily is shown with IGF-I as a model. **(D)** Folding nucleus of 1SS-DesDi single chain visualized as a proinsulin model. Initial pairing of B19-A20 disulfide in combination with the formation of two-helices (B9-B19 and A12-A20) are considered to be central events in the formation of folding nucleus. Dotted lines represent disordered regions. Larger circles represent the Asp^B10^ and Glu^A8^ substitutions. Key residues are highlighted by their sequence position [panel **(C, D)** modified from Hua et al. (22)].

Native insulin contains two chains, B (30 residues) and A (21 residues) (21). Its native structure contains three α-helices stabilized by two inter-chain disulfide bridges (cystines B7-A7 and B19-A20) and one intrachain bridge (A6-A11; **Figure 1A**) (23). Whereas chain combination between the isolated chains is inefficient (24), cellular biosynthesis exploits a single-chain precursor, proinsulin, wherein disulfide pairing is intramolecular (25). Human proinsulin contains a disordered 35-residue connecting domain (C domain) between Thr^B30^ and Gly^A1^. Efficiency of disulfide pairing in single-chain precursors can be augmented by shortening the C domain or deleting it entirely (26). The present peptide model contains a peptide bond between residues B28 and A1 (red bars in **Figure 1B**); the native Pro^B28^ is substituted by Lys to permit convenient enzymatic cleavage to a two-chain hormone (red in sequence N in **Figure 1B**) (27). The 49-residue synthetic precursor (designated “DesDi”) exhibits remarkable folding efficiency, enabling preparation of certain insulin analogs refractory to classical chain combination (27). In the 1SS model cystine B7-A7 (solvent exposed in native insulin) is pairwise substituted by Ser whereas cystine A6-A11 (buried in the core of native insulin) is pairwise substituted by Ala (18). Segmental α-helical propensity and solubility were augmented by acidic surface substitutions His^B10^→Asp (28, 29) and Thr^A8^→Glu (30). The seven amino-acid substitutions in the parent 1SS peptide (positions B7, B10, B28, A6-A8 and A11) are highlighted in red in **Figure 1B** (entry 1SS-WT). Relative to an homologous two-chain peptide model of the corresponding 1SS IGF-I folding intermediate (**Figure 1C**) (22), we anticipated that the 1SS DesDi-based single-chain model would exhibit a conformational equilibrium biased toward a collapsed state (**Figure 1D**).

**Figure 2:**
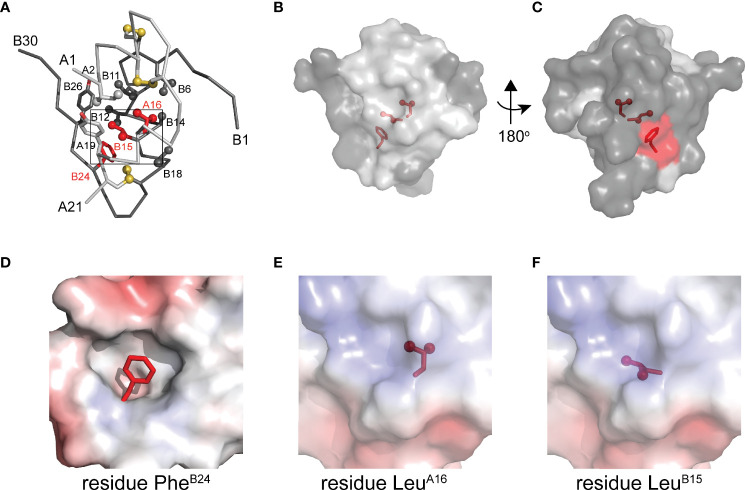
Native structure of insulin and sites of present MIDY mutations. **(A)** ribbon diagram of insulin monomer (PDB entry 4INS). Side chains of key residues are shown as sticks with methyl groups and sulfur atoms in disulfide bonds are shown as spheres (one third van der Waals radii). Clinical mutations under the study is shown in red and all other side chains follow the color code: A chain (light gray), B chain (dark gray). **(B)** Surface representation of insulin monomer (transparency set to 40%) showing the side chains of Phe^B24^, Leu^B15^ and Leu^A16^ (sticks shown as in panel A) within the core of the molecule. The surface of these residues is shown in red. **(C)** A view after rotating the molecule in panel B by 180° through y-axis. **(D–F)** Respective close-up views of the environments of Phe^B24^ (crevice), Leu^A16^ (core) and Leu^B15^ (core). In these panels the highlighted side chain is shown in red. These electrostatic surfaces were calculated in absence of indicated side chain.

To connect our model to patient phenotypes, three MIDY mutations were introduced into the 1SS peptide model (**Figure 1B**). Two (Pro^B15^ and Pro^A16^) are associated with neonatal-onset DM (31, 32); the third (Ser^B24^) is associated with onset in early adulthood (33). The structural environments of these conserved side chains are shown in **Figure 2A**. Whereas the side chains of Leu^B15^ and Leu^A16^ are buried in the hydrophobic core (**Figures 2B, C, E, F**), the aromatic side chain of Phe^B24^ packs within a crevice overlying internal cystine B19-A20 such that one side of the aromatic ring is exposed to solvent (**Figures 2B–D**). Initial characterization of these peptides was described in our companion article (18). Whereas the Pro substitutions introduced marked perturbations in folding efficiency in a mini-proinsulin [“DesDi”, (27)], Ser^B24^ was well tolerated. Synthetic yields mirrored residual α-helix contents (as inferred from far-UV circular dichroism; CD) in the corresponding 1SS peptides (18). A further correlation was observed between these properties and a pertinent pathogenetic process: extent of ER stress induced in a human cell line (HEK 293T) on transient expression of the corresponding mutant proinsulins. The coherence of these correlations (18) motivated this companion study wherein NMR spectroscopy provides a residue-specific view.

In this companion study we employ two-dimensional ^1^H- and [^1^H, ^13^C]-NMR methods to interrogate the 1SS peptide models in relation to the native insulin. Analysis of main-chain ^1^H and ^13^C chemical shifts in the parent peptide (34–36) provided evidence for nativelike nascent α-helices in the B domain (residues B9-B19) and A domain (A12-A20), together in accordance with CD-defined α-helix content (18). Although chemical-shift dispersion in this and the variant partial folds is more limited than in the spectrum of a native insulin monomer (37), key side-chain resonance assignments were verified by site-specific ^13^C (and ^13^C, ^15^N) labeling, using selective labeled amino-acid precursors in chemical peptide synthesis. Analysis of signature chemical shifts and framework nuclear Overhauser effects (NOEs) provides evidence for a nativelike folding nucleus in the parent 1SS peptide that is dependent on maintenance of the B19-A20 disulfide bridge. The clinical mutations perturb this nascent structure in the order Ser^B24^ (least perturbed) >> Pro^A16^ > Pro^B15^ (most perturbed). Together, these findings suggest that the DesDi-based 1SS model will provide a general platform for comparative biophysical studies of that subset of MIDY mutations that perturb initial closure of cystine B19-A20 in proinsulin biosynthesis in pancreatic β-cells.

## Materials and Methods

### Solid-Phase Peptide Synthesis

Peptides were synthesized either with an ABI 433A Peptide Synthesizer (Applied Biosystems) or Tribute 2-Channel peptide synthesizer (Gyros Protein technologies) using a preprogrammed solid-phase fluorenylmethyloxycarbonyl chloride (Fmoc) protocol designed for standard 0.1mmol scale syntheses. ABI protocols consist of the following modules: cycle-1 [d], cycle-2 [aibde], cycle-3 with number of repetitions equal #aa-1 [afgbde], cycle-“#aa+2” [ffbdc], where #aa is a number of amino acids in the sequence. Automated couplings utilized diisopropylcarbodiimide (DIC)/6-Cl-hydroxybenzotriazole (6-Cl-HOBt) in *N*-methyl pyrrolidone (NMP) whereas Fmoc deprotections used 20% piperidine in NMP. α-Carboxyl-protected Asp was used in place of Asn in all syntheses of DesDi analogs to accommodate the use of ChemMatrix^®^ Rink-Amide resin (loading = 0.46mmol/g). The Tribute peptide synthesizer used heating protocols: coupling was done at 6 min at 60°C except for Cys/His (2 min at 25°C, then 5 min at 60 °C) and Arg (20 min at 25 °C, then 5 min at 60°C); deprotection was done twice (30 sec at 50°C, then 3 min at 50°C). Reagent conditions were otherwise similar to ABI protocols except that DMF was used as solvent and choice of the resin was H-Asn(Trt)-HMPB-ChemMatrix^®^ resin. Peptides were cleaved with a trifluoroacetic acid (TFA) cocktail (2.5% vol/vol of each: β-mercaptoethanol, triisopropylsilane, anisole, and water) followed by ether precipitation.

### Folding and Purification of N and N* DesDi Analogs

Crude peptides from ether precipitation were dissolved in glycine buffer (20mM glycine and 2mM cysteine hydrochloride, pH 10.5) to a final peptide concentration of 0.1mM. The pH of this solution was readjusted to 10.5 to account for traces of residual TFA present in lyophilized peptides. This solution was then stirred while open to air at 4°C until reaction completion (usually overnight). The pH of the solution was then lowered to ~2.0 with 5N HCl to neutralize the folding reaction. Folded peptide was then purified by preparative rp-HPLC with a 60 min 20→40% acetonitrile elution gradient (10-15mL/min flow rate) run by a Waters rp-HPLC (Milford, MA) equipped with a Waters Model 600 Controller, Waters 2487 Dual Wavelength Detector, ProStar Model 701 Fraction Collector, Kipp & Zonen BD41 chart recorder, and a Luna 10μm C8(2) 100A AXIA (250 × 21.2mm) column. Fractions containing clean peptide were pooled and lyophilized. Purity of the materials was confirmed by LCQ Advantage Ion Trap Mass Spectrometer System coupled to an Agilent 1100 Series HPLC system utilizing a TARGA C8 5-μm (250 mm x 4.6 mm) column and a 35 min 25→50% acetonitrile elution gradient (1mL/min flow rate). See **Table S1** for LCMS retention times and mass verification.

### Synthesis of Isotopically Labeled Peptides

Isotopically labeled 1SS and control peptides were prepared on 0.05 mmol scales. N* was assembled in entirety as a single batch whereas the 1SS peptides were assembled as a single batch (0.1mmol) through residue B25. At that point, half of the 1SS resin was used to complete assembly of the 1SS-Ser^B24^ analog. The remaining resin was used to complete the 1SS synthesis. Coupling of isotopically labeled amino acids (Cambridge Isotopes Inc., Tewksbury, MA) was performed manually using a single 0.3mmol mixture consisting of equivalent amounts of labeled Fmoc amino acid, 1-[Bis(dimethylamino)methylene]-1*H*-1,2,3-triazolo[4,5-*b*]pyridinium 3-oxid hexafluorophosphate (HATU), and *N,N*-diisopropylethylamine (DIEA) reacted with each individual batch of resin (2.25X overall excess). The same automated synthesis protocol used for unlabeled peptide syntheses was then used for the addition of subsequent unlabeled amino acids to the labeled peptide assembly. The following labeled amino acids were used (residual positions given in blue in **Scheme S1**): Fmoc-Gly[^13^C_2_, ^15^N]-OH, Fmoc-Leu[^13^C_6_, ^15^N]-OH, Fmoc-Ile[^13^C_6_, ^15^N]-OH, Fmoc-Val[^13^C_5_, ^15^N]-OH, and Fmoc-Tyr[^13^C_9_, ^15^N]-OH. The peptides were cleaved from the resin by treatment with TFA cocktail as described above. The folding and purification of these isotope labeled 1SS peptides followed essentially same protocol as described in the companion article (18). Purity of the materials was confirmed by LC-MS with an Agilent 1260 Infinity/6120 Quadrupole instrument utilizing a Kinetex C8 2.6-μm 100A (75 mm x 2.1 mm) column and a 10 min 10-80% acetonitrile elution gradient (1mL/min flow rate) (See **Figures S1-S3**).

### Purification of Clinical Analogs

Wild-type insulin and insulin *lispro* were purified from U-100 pharmaceutical formulations of Humulin^®^ and Humalog^®^ (Eli Lilly and Co.), respectively, using preparative RP-HPLC (C4 10μm 250×20mm Proto 300 Column; Higgins Analytical, Inc.) utilizing buffer A (0.1% TFA in H_2_O) and a 10-min elution gradient of 20→70% buffer B (0.1% TFA in acetonitrile). Following lyophilization of the collected protein fraction, purity was verified using analytical HPLC (TARGA C8 5-μm [250 mm x 4.6 mm]; Higgins Analytical, Inc.) with a 35-min elution gradient of 25→50% buffer B; molar mass was verified with an Applied Biosystems 4700 Proteomics Analyzer utilizing MALDI-TOF in reflector mode. chromatographic retention times and mass measurements for these clinical analogs are given in **Table S1**.

### Proinsulin Constructs

Plasmids expressing full-length human proinsulin or variants were constructed by polymerase chain reaction (PCR). Mutations in proinsulin were introduced using QuikChange™ (Stratagene). Constructions were verified by DNA sequencing.

### NMR Spectroscopy

^1^H NMR spectra were acquired at a proton frequency of 700 MHz at pD 7.4 (direct meter reading) at 35°C. ^1^H-^13^C heteronuclear single-quantum coherence (HSQC) spectra were acquired at natural abundance as described. The spectra were obtained at ^13^C frequency of 176 MHz at a constant temperature of 308 K using the “hsqcetgp” Bruker pulse sequence as described by the manufacturer. Aliphatic ^1^H-^13^C HSQCs were acquired with FID size 2048 x 128, 800 scans, 1.0 sec relaxation delay, sweep widths 11 ppm (^1^H) and 70 ppm (^13^C) with offset 4.7 and 40 ppm for the ^1^H and ^13^C dimension, respectively. Similar parameters were used to acquire aromatic ^1^H-^13^C HSQC, except with sweep widths of 40 ppm and 125 ppm offset in ^13^C dimension. Data were processed with Topspin 4.0.6 (Bruker Biospin) and analyzed with Sparky software (38) using a 90° shifted-sine window function to a total of 2048 × 1024 data points (F2 × F1), followed by automated baseline- and phase correction. All NMR data were acquired using a BRUKER 700 MHz spectrometer equipped with quadruple [^1^H, ^19^F, ^13^C, ^15^N]-resonance liquid-helium-cooled cryoprobe.

### Secondary Structure Analysis

Protein secondary structure was inferred from selected ^1^H and ^13^C secondary chemical shifts (^1^H_N_, ^1^H_α_, ^13^C_α_ and ^13^C_β_) as described (35, 39, 40). In such algorithms ^13^C_α_ and ^1^H_α_ chemical shifts distinguish α-helix from β-strand or random coil (41) whereas ^13^C_β_ secondary shifts are more sensitive to β-strand. These chemical shifts in the parent 1SS model were assigned on the basis of 2D ^1^H-^1^H NOESY, TOCSY, DQF-COSY in D_2_O and H_2_O (10% D_2_O) and natural abundance ^1^H-^13^C HSQC spectra. Corresponding secondary shifts were extracted from observed chemical shifts (Δ = δ_obs_ - δ_coil_). Secondary structural elements were predicted by TALOSplus (34–36).

### Molecular Modeling

Structural ensembles were calculated by simulated annealing using *XPLOR-NIH* (42–44). The models of the one-disulfide proinsulin and one-disulfide DesDi intermediates (containing cystine B19-A20) were generated using distance restraints pertaining to residues A16-A21 and B15-B26 as observed in an engineered proinsulin (45) or an engineered insulin monomer (46). To allow for protein flexibility in these partial folds, upper bounds on long-range distance restraints were increased by 3 Å relative to NMR-derived upper bounds obtained in prior studies of insulin and proinsulin (45, 46).

## Results

**Figure 3:**
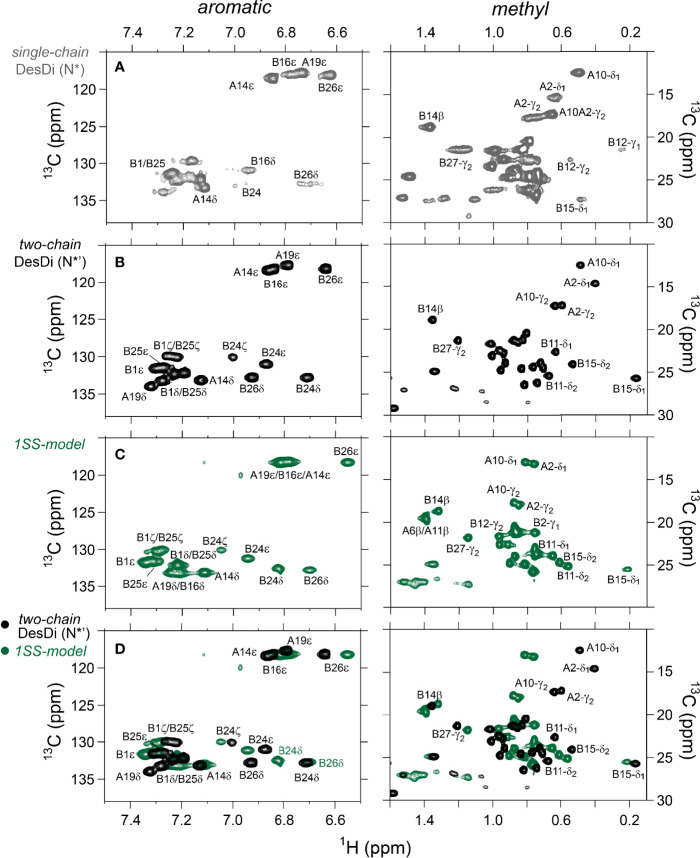
Nature abundance ^1^H,^13^C HSQC NMR spectra of DesDi analogs in aromatic (*left panel*) and methyl region (*right panel*). **(A)** single-chain DesDi (N*); **(B)** two-chain DesDi (N*’); **(C)** single-chain 1SS-DesDi model (*green*) and **(D)** the ^1^H-^13^C HSQC spectral overlay of two-chain DesDi (N*’, *black*) and 1SS-model (*green*). Spectra were acquired at a ^1^H frequency of 700 MHz at pD 7.4 (direct meter reading) and at 35°C in D_2_O. Selected resonance assignments are as indicated.

**Figure 4:**
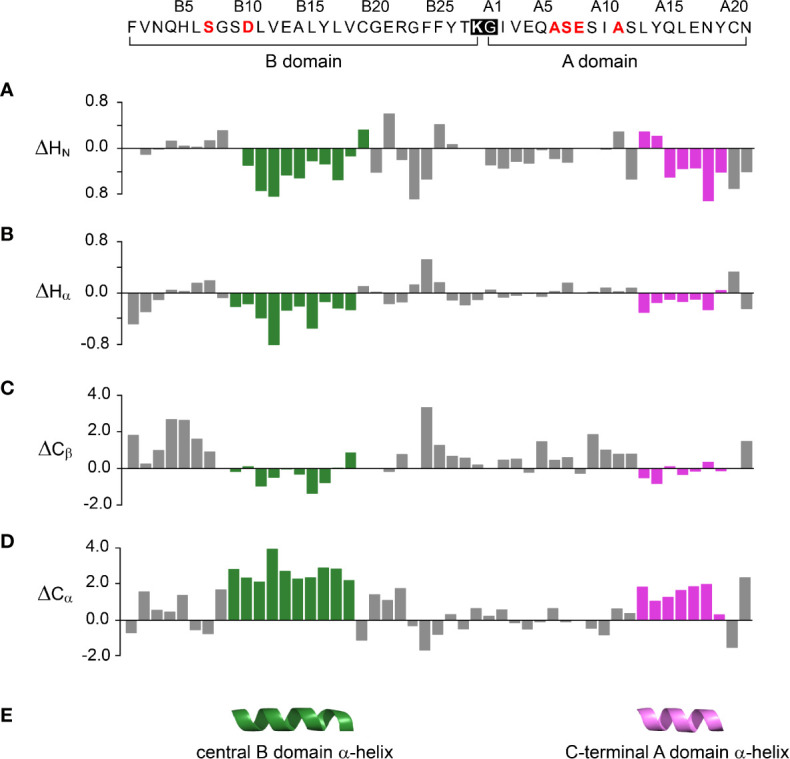
Chemical shift index (CSI) and inferred secondary structure of parent peptide model. *Top*, 49-residue polypeptide sequence with six amino-acid substitutions in red. DesDi’s engineered junction between Lys^B28^ (bold) and Gly^A1^ (27) is highlighted within black box. The secondary shift was defined as the difference of observed chemical shift and random coil shift (Δ = δ_obs_ - δ_coil_). Positive (negative) values indicate that the observed chemical shift is downfield (upfield) of expected random-coil frequency (34, 35). **(A–D)** CSI-related secondary chemical shifts by residue: **(A)** amide protons (ΔH_N_); **(B)** α-protons (ΔH_α_); **(C)** β-carbons (ΔC_β_); **(D)** α-carbons (ΔC_α_). **(E)** Inferred elements of secondary structure as predicted by *TALOSplus* (34–36): central B-domain α-helix (residues B9-B18) and C-terminal A-domain α-helix (residues A13-A19). Corresponding residue-specific secondary shifts are given in **Table S9**.

One-dimensional ^1^H-NMR spectra of the 1SS peptide model and its variants were presented in our companion study (18) in relation to spectra of the native state (provided as **Figure S4** for convenience of the reader). Molecular properties of these peptides are summarized in **Table S1**. Although the 1D spectra were in overall accordance with trends in synthetic yield, CD deconvolution and redox stability (18), interpretation was limited by the small number of resolved features. To circumvent this limitation, 2D homonuclear and 2D ^1^H-^13^C HSQC NMR spectra were obtained at natural abundance. Analysis was undertaken in reference to baseline HSQC spectra of native DesDi (as the single-chain precursor and as cleaved two-chain hormone analog; **Figures 3A, B****)**. Near-complete assignment of ^1^H_N_, ^1^H_α_, ^13^C_α_ and ^13^C_β_ resonances in the parent 1SS peptide model enabled mapping of secondary structure based on pattern of secondary chemical shifts (**Figure 4**) (34–36). α-Helical segments comprise residues B9-B18 and A13-A19 (**Figure 4E**), a subset of native secondary structure. Chemical shifts (referenced below) and estimates of chemical-shift dispersion are tabulated in **Tables S2****–****S9**.

^1^H-^13^C HSQC spectra provide correlations between a ^13^C atom and an attached proton (^1^H) *via* a one-bond J-coupling (47, 48). Initial spectra were obtained at 35 °C. Key resonance assignments in the parent 1SS peptide and 1SS-Ser^B24^ variant were verified by site-specific ^13^C labeling of residues Val^B12^, Leu^B15^, Gly^B23^, Phe^B24^, Tyr^B26^ and Ile^A2^ (**Scheme S1** and **Figure S5**). In each figure aromatic 2D spectra are shown at left, and aliphatic spectra at right. The spectrum of the native state is better illustrated by two-chain [Asp^B10^, Glu^A8^]-DesDi-insulin (**Figure 3B**; black) than its single-chain precursor (**Figure 3A**; gray) due to selected resonance broadening in the latter spectrum; such broadening may reflect partial dimerization (stronger in single-chain analogs) and/or conformational exchange intermediate on the timescale of NMR chemical shifts. The baseline 2D ^1^H-^13^C HSQC spectrum of two-chain [Asp^B10^, Glu^A8^]-DesDi-insulin is notable for its resolution of many individual spin systems; selected resonance assignments are provided in **Figures 3A, B**. Of particular interest are the aromatic resonance of Phe^B24^ and Tyr^B26^, which pack against the central B-chain α-helix and influence the chemical shifts (via their aromatic ring currents) of the side chains of Leu^B11^ and Leu^B15^. These respective upfield aromatic and upfield methyl resonances provide markers of the native B-chain supersecondary structure (49, 50). The upfield chemical shifts of Ile^A2^ by contrast reflects A-chain supersecondary structure (in particular the aromatic ring current of Tyr^A19^); that of Ile^A10^ reflects inter-chain packing of His^B5^ (51, 52). The latter structural features (and their NMR signatures) are reinforced by cystines A6-A11 and B7-A7 in native insulin.

**Figure 5:**
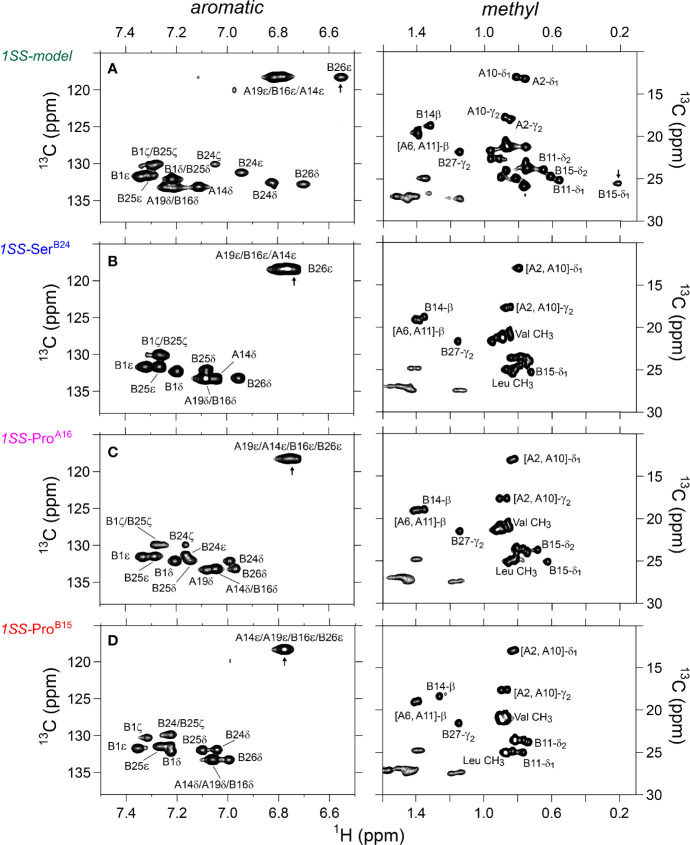
Nature abundance ^1^H-^13^C HSQC NMR spectra of single-chain 1SS-DesDi analogs in aromatic (*left panel*) and methyl region (*right panel*). **(A)** single-chain 1SS model; **(B)** single-chain 1SS-Ser^B24^ analog. **(C)** single-chain 1SS-Pro^A16^ analog and **(D)** single-chain 1SS-Pro^B15^ analog. Spectra were acquired at a ^1^H NMR frequency of 700 MHz at pD 7.4 (direct meter reading) and 35°C in D_2_O. Selected resonance assignments are as indicated.

The 2D ^1^H-^13^C HSQC spectrum of the parent 1SS model (**Figure 3C**; green) is remarkable for preservation of some upfield shifts (Phe^B24^, Tyr^B26^, Leu^B11^ and Leu^B15^) but not others (Ile^A2^ and Ile^A10^). The former subset of nativelike chemical shifts indicates maintenance of B-chain secondary structure flanking Cys^B19^ (and therefore the B19-A20 disulfide bridge) whereas the latter attenuation of structure-dependent secondary shifts indicates destabilization of long-range structure involved the A2-A10 segment in accordance with removal of the B7-A7 and A6-A11 disulfide bridges (50, 53, 54). This combination of preserved and attenuated chemical shifts in the spectrum of the parent 1SS model is illustrated by overlay of its spectrum (green) and that of two-chain [Asp^B10^, Glu^A8^]-DesDi-insulin (black) in **Figure 3D**. The spectrum of the parent 1SS peptide in turn provides a baseline for comparative analysis of the MIDY-derived variants (**Figure 5**). Chemical-shift dispersion is markedly attenuated in each of the variants (**Figures 5B–D**), most markedly in 1SS-Pro^B15^ (**Figure 5D**). In the case of 1SS-Ser^B24^ (**Figure 5B**) such attenuation may reflect both loss of ordered structure and removal of the Phe^B24^ aromatic ring current (52), potentially a confounding issue (the same issue pertains to Ser^B24^-insulin analogs with native pairing; **Figure S6**). A subtle trend is observed in the ^1^H_ϵ_ chemical shift of Tyr^B26^ (arrows in **Figures 5B–D**) in which slight upfield shifts are observed in two case (1SS-Ser^B24^ > 1SS-Pro^A16^), but not observed in 1SS-Pro^B15^. This trend is shown in an enlargement of spectra in **Figure S7**.

**Figure 6:**
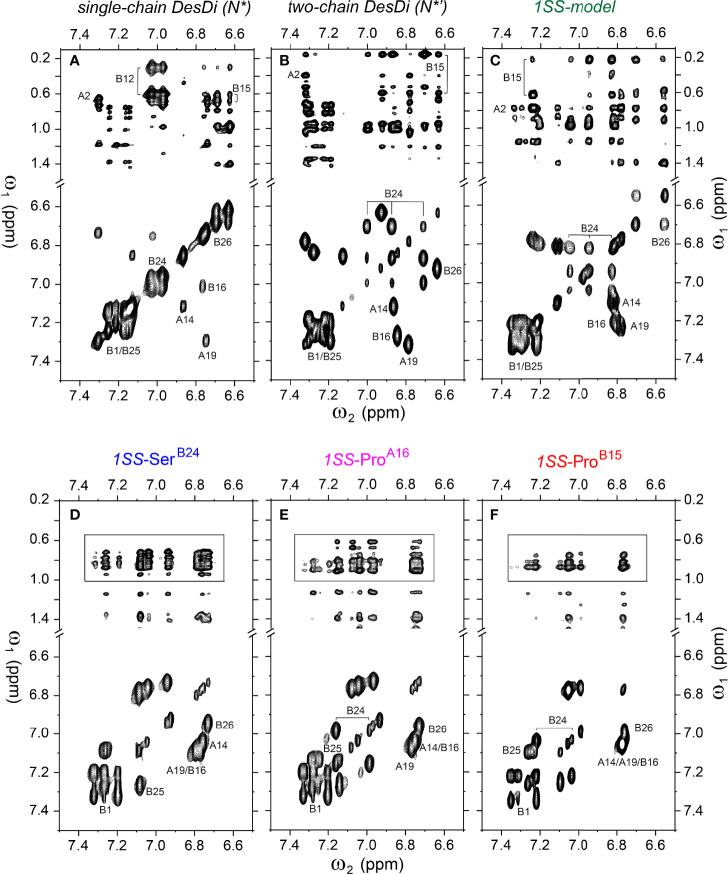
Homonuclear 2D-NMR spectra: NOESY spectra (mixing time 150 ms) showing NOEs from aromatic protons to methyl protons (*top panel*) and TOCSY spectra (mixing time 55 ms) showing aromatic resonance correlation (*bottom panel*). **(A)** single-chain DesDi (N*); **(B)** two-chain DesDi (N*’); **(C)** single-chain DesDi 1SS model; **(D)** single-chain 1SS-Ser^B24^ analog; **(E)** single-chain 1SS*-*Pro^A16^ analog and **(F)** single-chain 1SS-Pro^B15^ analog. The boxes in panel D-F indicate spectral expansion for detailed analysis. Spectra were acquired at pD 7.4 (direct meter reading) and 35°C in D_2_O.

**Figure 7:**
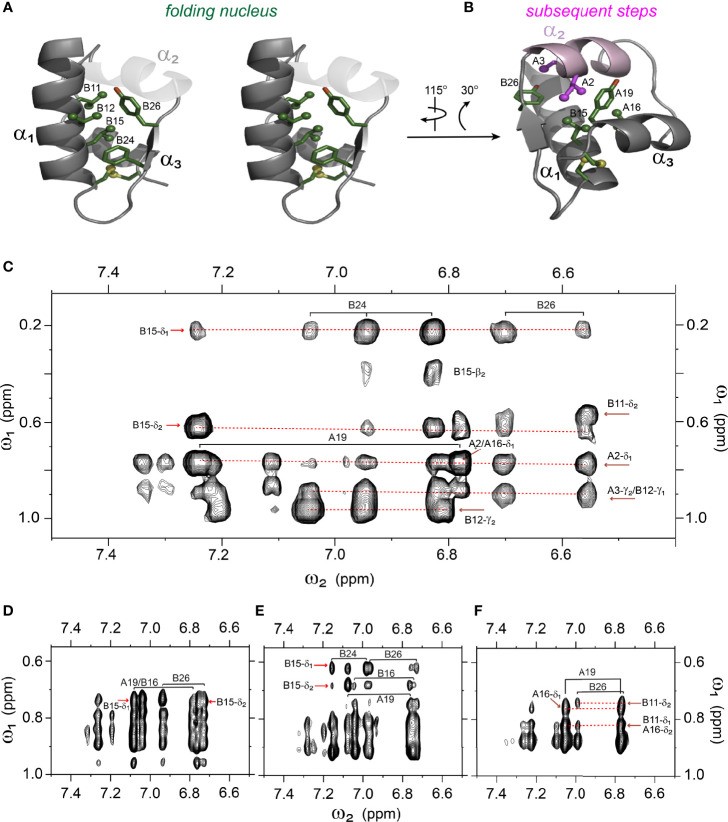
Spectral expansion of 2D-NOESY revealing signature long-range NOEs in the variants of single-chain 1SS*-*DesDi. **(A)** stereo view of the ribbon structure of single-chain DesDi (PDB entry 7JP3). The side-chains of indicated residues assembled a hydrophobic cluster that functions as a folding nucleus. **(B)** the ribbon structure of single-chain DesDi showing subsequent steps of insulin folding. **(C)** NOESY expansion of single-chain DesDi 1SS model. **(D)** single-chain 1SS*-*Ser^B24^ analog. **(E)** single-chain 1SS-Pro^A16^ analog and **(F)** single-chain 1SS*-*Pro^B15^ analog. All spectra were acquired at pD 7.4 (direct meter reading) and at 35°C in D_2_O.

Analysis of ^1^H-^13^C chemical shifts was extended by 2D ^1^H-^1^H NOE spectroscopy (NOESY). Of particular interest are inter-proton NOEs (reflecting distances < 5 Å) between aromatic and aliphatic side chains. Such NOEs are prominent in the spectrum of native insulin (upper panel of **Figures 6A, B**), shown in relation to the corresponding TOCSY (total correlation spectroscopy) spectra of aromatic spin systems (lower panel). These inter-residue NOEs in part retained in the NOESY spectrum of the parent 1SS peptide (upper panel in **Figure 6C**). Particularly notable is the retention of close contacts between Phe^B24^ and Tyr^B26^ and the methyl groups of Leu^B15^, resolved due to native-like chemical-shift dispersion. A subtle feature is observed in the aromatic TOCSY spectra: the spin systems of Tyr^B16^ and Tyr^A19^, downfield of the mobile and solvent-exposed side chain of Tyr^A14^ in native insulin (lower panels of **Figures 6A, B**), is retained in attenuated form in the TOCSY spectrum of 1SS peptide (lower panel of **Figure 6C**). NOEs between aromatic and aliphatic protons are observed in the spectra of the variants, but with decreased dispersion (inset boxes in **Figures 6D–F**); in the case of 1SS-Pro^B15^, the overall integrated cross-peak envelope intensity is reduced (**Figure 6F**). Although as expected the aromatic spin system of Phe^B24^ is absent in the TOCSY spectrum of 1SS-Ser^B24^ (lower panel of **Figure 6D**), subtle upfield shifts of Phe^B24^ are retained in 1SS-Pro^A16^ and 1SS-Pro^B15^. These trends are shown in expanded form in **Figure S8**. We imagine that the latter conformational ensembles contain a minor fraction of compact substates with long-range contacts, which nonetheless are less populated than in the parent 1SS peptide. This interpretation is supported by more detailed examination of these NOESY regions (**Figures 7C–F**) in relation to the structural relationships in native insulin (**Figures 7A, B**). Aromatic-methyl NOEs in the parent 1SS model are shown in expanded form in **Figure 7C**; long-range contacts are prominent in the neighborhood of cystine B19-A20, proposed to constitute the specific folding nucleus of proinsulin (green side chains in **Figure 7A**) (22). Also observed are long-range NOEs from Tyr^B26^ to the methyl groups of Ile^A2^ and Val^A3^, presumably reinforced by the B28-A1 peptide bond in the DesDi framework and foreshadowing subsequent steps in A-domain segmental folding associated with pairing of the remaining two cystines. This subset of these nativelike long-range NOEs can be resolved in the variants despite their attenuated chemical-shift dispersion (**Figures 7D–F**).

**Figure 8:**
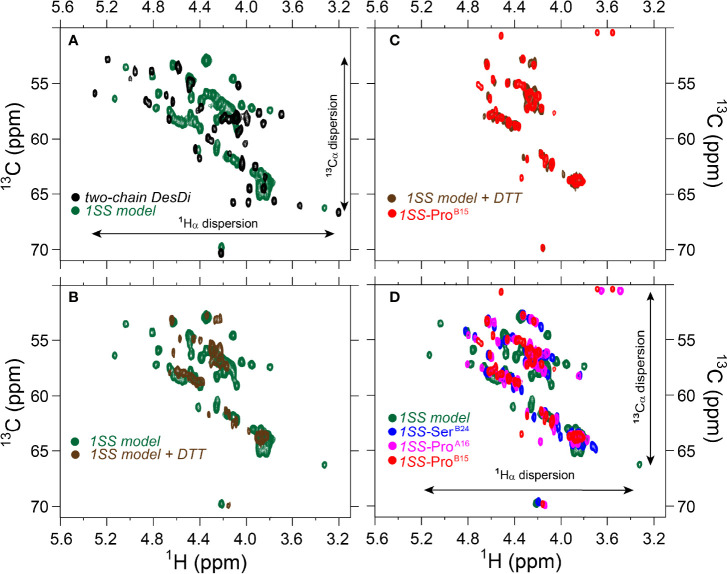
Comparison of nature abundance ^1^H-^13^C HSQC spectra of single-chain DesDi analogs in ^1^H_α_/^13^C_α_ region. **(A)** ^1^H-^13^C HSQC spectral overlay of two-chain DesDi (N*’, *black*) and single-chain DesDi 1SS model (*green*); **(B)** ^1^H-^13^C HSQC spectral overlay of single-chain DesDi 1SS model (*green*) and 1SS model in 70 mM deuterated dithiothreitol (DTT) (*brown*); **(C)** spectral overlay of single-chain 1SS*-*Pro^B15^ analog (*red*) and *1SS-model* in 70 mM deuterated dithiothreitol (*brown*); **(D)** spectral overlay of single-chain DesDi 1SS model (*green*), single-chain 1SS*-*Ser^B24^ analog (*blue*), single-chain 1SS*-*Pro^A16^ analog (*magenta*) and single-chain 1SS*-*Pro^B15^ analog (*red*). Spectra were acquired at a ^1^H frequency of 700 MHz at pD 7.4 (direct meter reading) and at 35°C in D_2_O.

The above degree of organization in the nascent structure of the parent 1SS model is dependent on pairing of Cys^B19^ and Cys^A20^. Whereas overlay of the ^1^H-^13^C HSQC spectra of the parent 1SS peptide and two-chain [Asp^B10^, Glu^A8^]-DesDi-insulin reveals similar limits of dispersion (green versus black in **Figure 8A**; inset vertical and horizontal arrows), reduction of the 1SS disulfide bridge by deuterated dithiothreitol (to yield a linear peptide) led to loss of ^1^H-^13^C chemical-shift dispersion (green versus brown in **Figure 8B**). The reduced 1SS peptide exhibits limited dispersion, with pattern that is similar in detail to that of 1SS-Pro^B15^ (brown versus red in **Figure 8C**). Possible transient or nascent long-range interactions in the linear 1SS peptide have not been investigated. ^1^H-^13^C HSQC spectra of the four 1SS peptides are overlaid in **Figure 8D**; relative main-chain dispersions exhibit the qualitative trend: parent > Ser^B24^ > Pro^A16^ > Pro^B15^ in accordance with the findings above. This trend was made quantitative by detailed analysis of respective secondary shifts [in reference to tabulated random-coil values (55)] as shown in the four histograms in **Figure S11**. Reliance of ^1^H_α_/^13^C_α_ chemical shifts circumvents the confounding absence of the B24 ring current in 1SS-Ser^B24^ as these resonances are less influenced by aromatic ring currents (52, 56). The greater main-chain chemical-shift dispersion in 1SS-Ser^B24^ and 1SS-Pro^A16^ relative to 1SS-Pro^B15^ was accentuated by lowering the temperature from 35 to 10°C. Stacked plots of 1D ^1^H-NMR spectra are shown for each 1SS peptide as a function of temperature in the range 5-35°C (in steps of 5°C) in **Figure S9**. At lower temperatures spectra of the parent 1SS peptide, 1SS-Ser^B24^ and 1SS-Pro^A16^ exhibit conformational broadening of upfield aromatic and aliphatic features, suggesting slowing of conformational fluctuations into the millisecond regime characteristic of intermediate exchange on the time scale of ^1^H-NMR chemical shifts. This phenomenon is not observed in the spectrum of 1SS-Pro^B15^. Comparison of ^1^H_α_-^13^C_α_ cross peaks in respective HSQC spectra likewise highlights the anomalous temperature-independence of the 1SS-Pro^B15^ spectrum (**Figure S10**). This trend extends to the aromatic and methyl regions of the HSQC spectra (**Figure S12**). The chemical shift of Tyr^B26^ H_ϵ_ and a resolved methyl resonance in the reduced 1SS peptide are likewise independent of temperature (**Figures S12**, **S13**). We speculate that the anomalous NMR properties of 1SS-Pro^B15^, indicating loss of nascent structure relative to the other 1SS peptides, rationalizes this mutation’s essentially complete block to the folding of Pro^B15^-DesDi, even in the presence of stabilizing substitutions Asp^B10^ and Glu^A8^ [preceding article in this issue (18)].

## Discussion

The discovery of proinsulin by Steiner and colleagues in 1967 [(25, 57); for review, see (58)] solved a problem encountered in the chemical synthesis of insulin: inefficient specific disulfide pairing encountered in chain combination (24). Although the isolated A- and B chains of insulin contain sufficient information to specify the native structure (59), yield is reduced by competing off-pathway reactions, including formation of cyclic peptides and amyloid. Proinsulin is nonetheless itself difficult to fold. The majority of human cell lines in routine laboratory use do not efficiency fold proinsulin (4), leading to detectable disulfide isomers (3). The specialized folding environment of the β-cell ER is adapted to the biochemical demands of proinsulin biosynthesis, and yet even so physiological overexpression of the *INS* gene—as a compensatory response to peripheral insulin resistance (60)—can induce chronic ER stress and contribute to the progression of prediabetes and Type 2 DM (8). The growing collection of MIDY mutations in proinsulin associated with toxic misfolding leading to β-cell dysfunction (9) has motivated the hypothesis that insulin sequences, well conserved among vertebrates (23, 61), are entrenched at the edge of foldability (46). The marginal stability of insulin and proinsulin, relative to such classical model proteins as bovine pancreatic trypsin inhibitor and hen egg white lysozyme, is associated with qualitative differences in their respective refolding properties (62–67) (see also Supplemental Discussion).

In this and our companion study (18) we have introduced a single-chain peptide model of an early proinsulin folding intermediate. A framework (“DesDi”) was provided by an innovative mini-proinsulin containing a peptide bond between residues B28 and A1, with Pro^B28^ substituted by Lys to enable facile enzymatic cleavage to liberate an active insulin analog (27). The B28-A1 peptide bond enables successful oxidative folding of the 49-residue synthetic precursor even in the presence of mutations (such as Val^A16^) that otherwise block classical chain combination (68). The B28-A1 inter-chain tether in DesDi presumably favors a productive orientation between A- and B-domain folding determinants and limits off-pathway events. We further stabilized DesDi by enhancing the α-helical propensity of the central B-domain segment [His^B10^→Asp (29)] and N-terminal A-domain segment [Thr^A8^ →Glu (30)]. Increasing the net negative charge through these acidic substitutions would also be expected to enhance solubility and retard competing formation of amyloid (69). A one-disulfide model of an initial proinsulin folding intermediate was thus obtained by pairwise substitution of exposed cystine B7-A7 by Ser and internal cystine A6-A11 by Ala (18).

**Figure 9:**
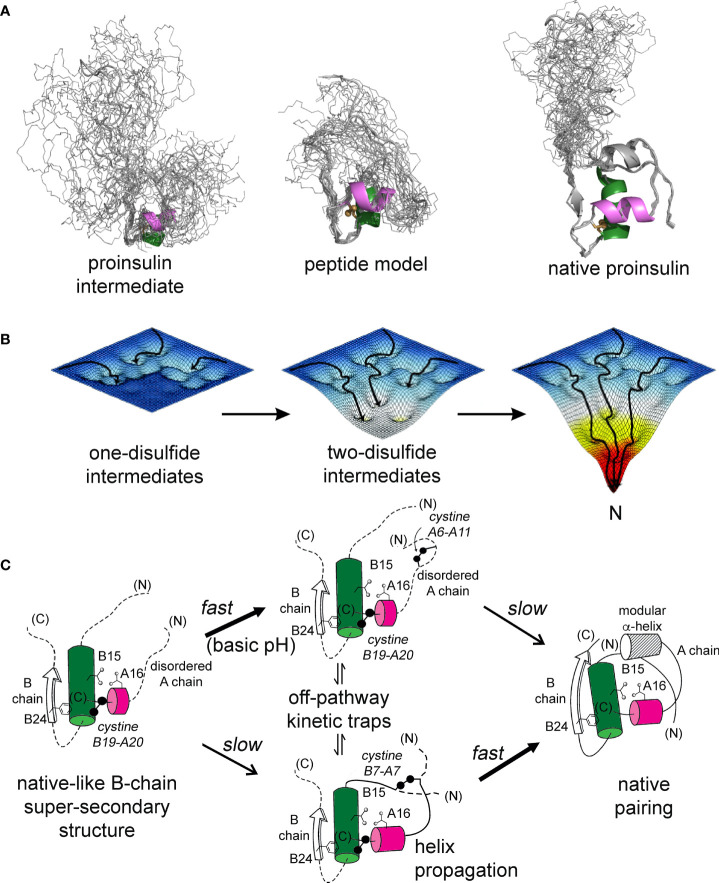
Proinsulin peptide models and hierarchical disulfide pathway. **(A)** Predicted structural ensembles for one-disulfide 86-residue proinsulin intermediate containing cystine B19-A20 (left) and corresponding 49-residue one-disulfide model (middle) relative to the solution structure of an engineered proinsulin monomer [PDB entry 2KQP; (45)] (right). In each ensemble α-helices are shown in green (central B domain) and magenta (C-terminal A domain). The simulated ensembles were generated by restrained molecular dynamics using XPLOR-NIH software (43, 44) and visualized by PyMol (https://sourceforge.net/projects/pymol/). **(B)** Free-energy landscapes as envisioned to govern proinsulin folding: disulfide pairing follows a sequence of folding trajectories on successively steeper landscapes. **(C)** Preferred pathway of disulfide pairing begins with cystine B19-A20 (left), directed by a nascent hydrophobic core formed by the central B-domain α-helix (residues B9–B19), part of the C-terminal B-chain β-strand (B24-B26) and part of the C-terminal A-domain α-helix (A16–A20). Alternative pathways mediate formation of successive disulfide bridges (middle panel) en route to the native state (right panel). This pathway is perturbed by diverse MIDY mutations. Nascent α-helices are color-coded as in **(A)**. Panel **(B)** is adapted from an image kindly provided by J. Williamson; Panel **(C)** is adapted from reference (70).

The present study builds on our foundational characterization of the 1SS peptide model to interrogate nascent structure by two-dimensional ^1^H and ^1^H-^13^C NMR spectroscopy. In accordance with prior NMR studies of a two-chain peptide model of a one-disulfide IGF-I folding intermediate (22), the parent 1SS model contains a subset of native secondary structure: central B-domain α-helix (residues B9-B19), C-terminal A-domain α-helix (A12-A20) and nascent β-strand (B24-B26). Molecular models of the parent model and the corresponding proinsulin intermediate are shown in **Figure 9A** in relation to the solution structure of an engineered proinsulin monomer (45). In these models cystine B19-A20 is integral to the hydrophobic mini-core formed at the confluence of the nascent elements of secondary structure. We envision that this nativelike subdomain represents the first organized nucleus in a series of successive folding landscapes (**Figure 9B**). Although disulfide chemistry in polypeptides can exhibit (especially at basic pH) complex patterns of native and non-native disulfide exchange and rearrangement, this structural perspective offers a simplified view of the predominant proinsulin folding scheme at neutral pH (**Figure 9C**). This scheme in principle provides a framework for interpreting clinical mutations that impair folding efficiency.

**Figure 10:**
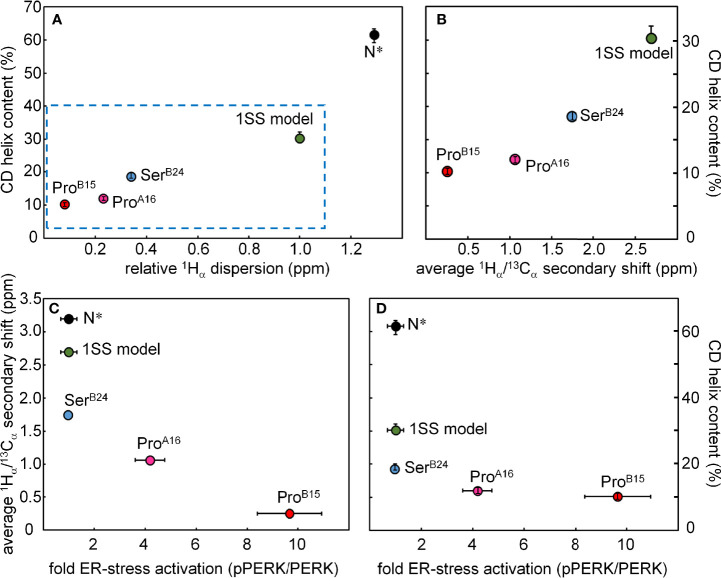
Plots showing correlations among biophysical features, NMR parameters and cellular ER-stress activation. **(A)** The plot shows the relationship between the relative ^1^H_α_ dispersion (X axis; ppm) CD-derived helix contents of DesDi variants (Y axis; %). N* analogs provide control data pertaining to the single-chain ground state stabilized by the three native disulfide bridges; sequences contain the same favorable substitutions at B10 and A8 as in the 1SS analogs. Relative ^1^H_α_ dispersion was defined by the difference of ^1^H_α_ chemical-shift dispersion of DesDi analogs and base line control (1SS model in 70 mM deuterated dithiothreitol). **(B)** Correlation between CD-derived helix contents (Y axis) with the average ^1^H_α_/^13^C_α_ secondary shift (X-axis). Average ^1^H_α_/^13^C_α_ secondary shift $\left(\bar{\Delta }\right)$ was calculated using equation $\bar{\Delta }=\sqrt{(\Delta_{B12}^{2}+\Delta_{B14}^{2}+\Delta_{B18}^{2})/3}.$ Δ_B12_, Δ_B14_ and Δ_B18_ are ^1^H_α_/^13^C_α_ secondary shift of residues Val^B12^, Ala^B14^ and Val^B18^, respectively. **(C, D)** Relationship between ER stress in cell-based assay and NMR/biophysical parameters. **(C)** Relationship between fold-change in ER stress (X axis; pPERK/PERK ratio) and ^1^H_α_/^13^C_α_ secondary shift (Y axis; ppm) among DesDi variants. **(D)** Relationship between ER stress and α-helix content of related DesDi variants (Y-axis; %).

Comparative NMR studies of the variant 1SS peptides suggest structural mechanisms of impaired foldability. In particular, patterns of chemical shifts and NOEs provide evidence of native-like tertiary structure in the neighborhood of cystine B19-A20 and its destabilization in the variant peptides in rank order Ser^B24^ >> Pro^A16^ > Pro^B15^ (least organized). Their respective ensembles of partial folds each exhibit a subset of nativelike long-range NOEs—presumably reflecting fractional occupancies of analogous molten-globule states that foreshadow native structural relationships—but with progressively more complete averaging of chemical shifts in this series (**Figure S14**). Among these 1SS peptides and native insulin, striking correlations are observed between CD-defined α-helix contents [in the same rank order (18)] and NMR parameters: mean ^1^H_α_ chemical-shift dispersion (**Figure 10A**) and average ^1^H_α_/^13^C_α_ main-chain secondary shifts (**Figure 10B**). The biological importance of these CD- and NMR-derived biophysical parameters is demonstrated by their further correlation with levels of ER stress induced by expression of the corresponding mutant proinsulin (**Figures 10C, D**) (18). Although each MIDY mutation alters an invariant framework residue—conserved among both vertebrate insulins and vertebrate insulin-like growth factors (23, 61, 71) —the less severe biophysical consequences of Phe^B24^→Ser in consistent with the delayed onset of DM in patients with this mutation (4, 33). Although Pro^B15^ is more profoundly perturbing than is Pro^A16^, each is associated with neonatal-onset DM (31, 32) and so must surpass the threshold for post-natal β-cell ER stress leading to the rapid progression of β-cell dysfunction and death (8).

A surprising aspect of the present NMR studies is extent to which the parent 1SS peptide retains native-like spectroscopic features. Such structure is lost on reduction of cystine B19-A20. We ascribe the nascent organization of the parent 1SS peptide to (a) the B28-A1 peptide bond, which orients flanking B- and A-domain segments, and (b) stabilizing α-helical substitutions Asp^B10^ and Glu^A8^. Although such extensive nascent structure would not be expected in a one-disulfide analog of proinsulin (86 residues), the richness of the 1SS NMR spectrum suggests that it would be of future interest to develop a biosynthetic expression system, so that uniform ^13^C and ^15^N isotopic labeling would enable application of powerful 3D/4D heteronuclear NMR methods (72, 73), including residual dipolar couplings (74). We foresee that such high-resolution analysis would enable comparison between the nascent structure and dynamics of the 1SS peptide and the classic globular structure of native insulin (23). Further, such complete ^1^H, ^13^C and ^15^N characterization would provide a rigorous platform for comparative studies of those MIDY mutations that perturb pairing of Cys^B19^ and Cys^A20^.

## Concluding remarks

The spectrum of diabetes-associated mutations in the insulin gene implicates diverse genotype-phenotype relationships (9, 75). These include not only mutations toxic misfolding of proinsulin with the ER (the present focus), but also those affecting upstream translocation of nascent preproinsulin (76, 77) and downstream trafficking, prohormone processing, and receptor binding (75). Each class of clinical mutations promises an opportunity to dissect respective molecular mechanisms critical to wild-type hormone biosynthesis and function. Because mutations may introduce mild, intermediate or severe biochemical perturbations, their comparative study may reveal quantitative thresholds of dysfunction associated with clinical features, such as age of diabetes onset or degree of genetic penetrance. Adult-onset Ser^B24^ represents a mild perturbation of folding efficiency whereas both neonatal-onset Pro^A16^ and Pro^B15^ mutations—albeit distinct in location and degree of structural perturbation—must be below the threshold of foldability required for β-cell viability. Extending the present approach to additional MIDY mutations may define molecular determinants of this threshold. The mutant proinsulin syndrome thus promises to provide an intriguing model to relate chemistry to biology in a prototypical disease of intracellular protein misfolding.

## Data Availability Statement

The original contributions presented in the study are included in the article/**Supplementary Material**. Further inquiries can be directed to the corresponding authors.

## Author Contributions

Chemical peptide syntheses were performed by BD, AZ, and RD. NMR studies were performed and interpreted by YY, MG, NW, and MW. Figures were prepared by YY, BD, MG, AZ, Y-SC and MW. The Supplementary Material was prepared by YY, BD, AZ and MW. All authors contributed to editing the manuscript with first draft prepared by MG and MW. Overall experimental design and oversight were provided by MW. All authors contributed to the article and approved the submitted version.

## Funding

This work was supported in part by grants to MW from the National Institutes of Health (R01 DK040949 and R01 DK069764). AZ was supported in part by Callibrium, LLC; the authors declare that this funder was not involved in the study design, collection, analysis, interpretation of data, the writing of this article or the decision to submit it for publication. MG was a Pre-doctoral Fellow of the National Institutes of Health (Medical Scientist Training Program 5T32GM007250-38 and Fellowship 1F30DK104618-01).

## Conflict of Interest

The authors declare that the research was conducted in the absence of any commercial or financial relationships that could be construed as a potential conflict of interest.

## Publisher’s Note

All claims expressed in this article are solely those of the authors and do not necessarily represent those of their affiliated organizations, or those of the publisher, the editors and the reviewers. Any product that may be evaluated in this article, or claim that may be made by its manufacturer, is not guaranteed or endorsed by the publisher.

## Glossary

**Table:**

MIDY	mutant *INS*-induced diabetes of the young
MODY	maturity-onset diabetes of the young
MPS	mutant proinsulin syndrome
PNDM	permanent neonatal diabetes mellitus
DesDi	single-chain insulin analog containing Lys^B28^ and lacking residues B29 and B30
N	DesDi insulin template with all three native disulfide bonds
N*	DesDi insulin control with solubility enhancements
[B19-A20]	DesDi variant based on N* retaining only the B19-A20 disulfide linkage
ER	endoplasmic reticulum
SCI	single-chain insulin
CD	circular dichroism
NMR	nuclear magnetic resonance
rp-HPLC	reverse-phase high-performance liquid chromatography
LC-MS	liquid chromatography-mass spectrometry
MALDI-TOF	matrix-assisted laser desorption ionization - time of flight
IGF	insulin-like growth factor
NOE	nuclear Overhauser enhancement
NOESY	nuclear Overhauser enhancement spectroscopy
TOCSY	total correlation spectroscopy
HSQC	heteronuclear single-quantum coherence. Insulin residues are denoted by residue type (in standard three-letter code) followed by the chain and position (e.g., Phe^B24^ designates a phenylalanine at the 24^th^ position of the B chain)
