# Supplementary material for: Peptide Model of the Mutant Proinsulin Syndrome. II. Nascent Structure and Biological Implications
Source: Front Endocrinol (Lausanne). 2022 Mar 1;13:821091. doi: 10.3389/fendo.2022.821091 (PMC8922542; doi:10.3389/fendo.2022.821091)
Supplement: Supplementary file 1 [file DataSheet_1.pdf]

# Supplemental Information

*for*

## Peptide Model of the Mutant Proinsulin Syndrome. II. Nascent Structure and Biological Implications

Yanwu Yang[†,*], Michael D. Glidden[†], Balamurugan Dhayalan[†], Alexander N. Zaykov, Yen-Shan Chen, Nalinda P. Wickramasinghe, Richard D. DiMarchi, & Michael A. Weiss[*]

### Table of Contents

Purpose of Supplement . . . . . 2
Supplemental Discussion . . . . . 2
Labeling Scheme S1 . . . . . 5
Figure S1 . . . . . 6
Figure S2 . . . . . 6
Figure S3 . . . . . 7
Figure S4 . . . . . 8
Figure S5 . . . . . 9
Figure S6 . . . . . 10
Figure S7 . . . . . 11
Figure S8 . . . . . 12
Figure S9 . . . . . 13
Figure S10 . . . . . 14
Figure S11 . . . . . 15
Figure S12 . . . . . 16
Figure S13 . . . . . 17
Figure S14 . . . . . 18
Table S1 . . . . . 20
Table S2 . . . . . 21
Table S3 . . . . . 22
Table S4 . . . . . 23
Table S5 . . . . . 24
Table S6 . . . . . 25
Table S7 . . . . . 25
Table S8 . . . . . 26
Table S9 . . . . . 27
References. . . . . . 28

## Purpose of Supplement

This Supplement outlines the synthetic scheme of selective incorporation of [$^{13}C$, $^{15}N$]-labeled amino acids into single-chain DesDi insulin analogs and single-chain “1SS” peptide models (**Scheme 1**) and provides 14 supplemental figures. The latter pertain to LC-MS profiles (**Figures S1-S3**), NMR spectroscopy (spectra in **Figures S4-S12**), NMR analysis (**Figure S13**) or schematic showing conformational averaging of NMR parameters (**Figure S14**). In addition, 9 supplemental tables are given. The first summarizes key properties of the present collection of single-chain insulin analogs (in the context of DesDi and Asp$^{B10}$, Glu$^{A8}$-DesDi frameworks; “native state”) and corresponding single-disulfide peptide models (“1SS”; **Table S1**). The folded states of single-chain DesDi and its stabilized Asp$^{B10}$, Glu$^{A8}$-variant are respectively denoted N and N*; 1SS peptides each contain substitutions His$^{B10}$→Asp and Thr$^{A8}$→Glu as described in our companion study (preceding article in this issue). These substitutions are intended to bias the conformational ensemble of the 1SS peptides toward nascent helix formation and to enhance solubility at neutral pH. Selected NMR resonance assignments and chemical shifts are given in **Tables S2-S9**.

Supplemental **Figures S4-S13** provide additional NMR spectra of DesDi analogs or 1SS peptides. Key NMR resonance assignments was confirmed by selectively labeled DesDi analogs (**Scheme S1** and **Figure S5**). Substitution of invariant Phe$^{B24}$ by MIDY mutant Ser$^{B24}$ leads to less-ordered structure as probed by 1D NMR spectra (**Figure S6**). Additional spectra of $^{1}$H-$^{13}$C-HSQC showing aromatic Cε and valine methyl regions for 1SS peptides (**Figure S7 and S8**). Temperature-dependence NMR studies are provided that probe folding at lower temperature as shown in **Figures S9-S10, S12**. Detailed analysis of secondary shifts is shown as the histograms in **Figure S11**. **Figure S13** provides a summary of chemical-shift perturbations with varying temperature for some aromatic and methyl protons. Assignments of selected NMR resonances and NMR parameters derived from chemical shifts are provided in **Table S2-S9**.

### Supplemental Discussion

*Folding Mechanisms of Classical Model Proteins*. The Anfinsen paradigm posits that protein folds are encoded by amino-acid sequences (1). This principle has been elaborated to highlight an interplay of temperature-dependent entropic and thermodynamic effects leading to funnel-shaped free-energy landscapes. The “new view” emphasizes biophysical determinants of folding efficiency (2) as an evolved property of polypeptides as a class of heteropolymers (3).

Oxidative protein folding is more complex than reversible two-state refolding but offered the experimental opportunity for chemical trapping studies (4). Unlike insulin, such classical disulfide-linked exploited in model studies typically exhibit marked thermodynamic stability. The literature is extensive and extends over the past three decades. Selected examples are provided by **bovine pancreatic trypsin inhibitor** (5-10), **human** and **hen egg-white lysozyme** (11-14), and **bovine pancreatic ribonuclease A** (15-18). Each of these models has attracted a community of investigators.

Despite its biomedical importance, the insulin superfamily is less well-studied, in large part because of technical challenges, but key insights have nonetheless emerged from this and other laboratories: (19-23). The present study (and its companion in this issue: (24)) build on this foundation. A general feature observed in studies of the oxidative folding of insulin-related polypeptides and its equilibrium models is stepwise acquisition of structure with success disulfide

pairing. Folding may be viewed as trajectories on a series of ever-steeper funnels, each labeled by disulfide pairing scheme (25).

The stepwise paradigm stands in contrast to the paradigm that disulfide bridges *reflect* native structure but do not *direct* such structure. For example, BPTI contains four disulfide bridges and yet adopts a nativelike conformation (able to inhibit trypsin) in the presence of only one or two such bridges (8, 9). Initial pairing of any of several potential disulfide bridges can stabilize a similar folding nucleus (**Reprinted Figure** from (26)), which corresponds to the native state's β-sheet-rich amide-proton exchange core (27). Unlike internal cystine B19-A20 in the folding of proinsulin and in the present models of this one-disulfide intermediate, the cystine in the 1SS BPTI analogs is peripheral to the ordered subdomain. The cross-link is proposed to destabilize competing folds and not to stabilize the β-sheet domain directly. A variety of NMR methods have been exploited to characterize the dynamics of this and related equilibrium models of BPTI intermediates. (see also caption to **Figure S14**).

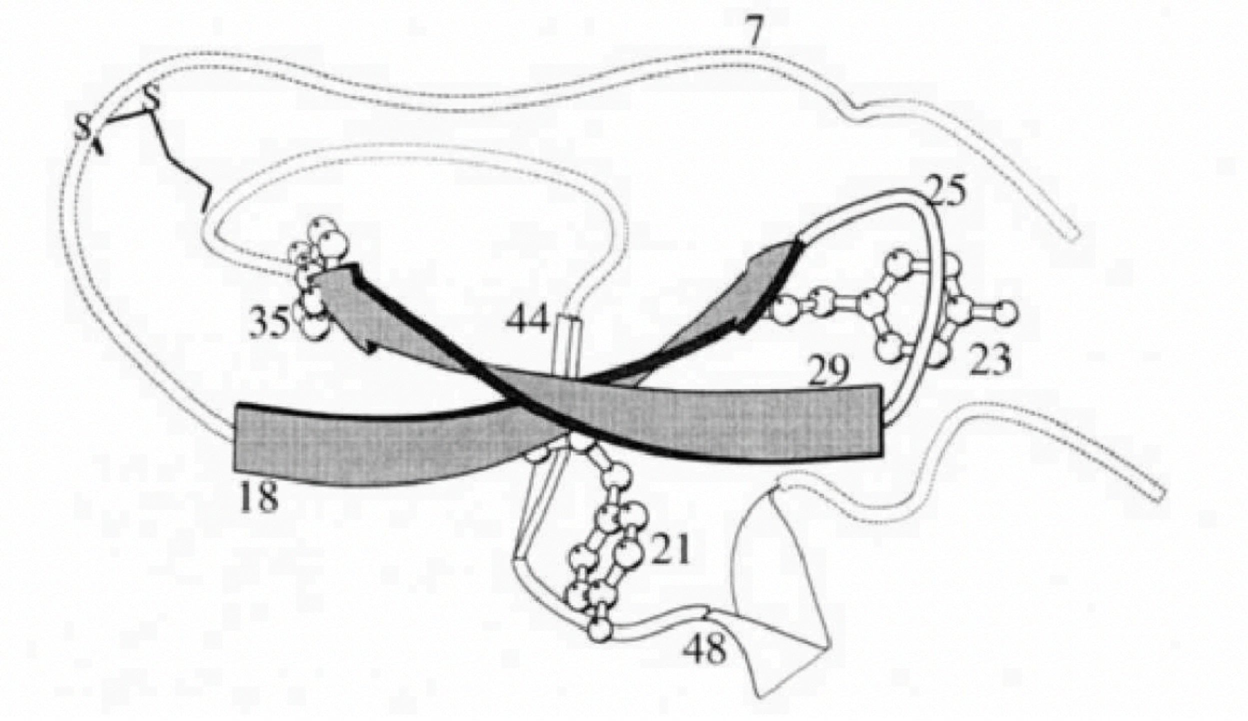


**REPRINTED FROM (26) (FIGURE 8).** Diagram of native BPTI structure. In [ 14—38]$_{Abu}$. the darkened antiparallel strands of β-sheet are native-like. Also in [l4-38]$_{Abu}$, residues 44—51, shown as a white ribbon drawn with solid lines, fluctuate between a major, native-like conformation and a more disordered one, while the sequences drawn with dotted lines are highly mobile and fluctuate between two or three slowly interconverting conformations. In [14—38]$_{Abu}$, the main hydrophobic contacts involve the side chains of Tyr 21, 23, and 35. shown with ball-and-stick. The figure was generated using the program MOLSCRIPT.

Although not in themselves intermediates, such BPTI analogs are less stable than the native state. After the first disulfide bridge (any one of several possibilities), subsequent disulfide bridges in BPTI thus stabilize the folded state *after it has already reached*, rather than directing folding by reshaping the shape of a free-energy landscape. Unlike BPTI, other proteins may exhibit partially folded disulfide intermediates: as in proinsulin, the native is reached only once all cystines are

paired. In such examples the disulfide bridges *direct* folding toward the ground state by stabilizing the folded state while also destabilizing non-native or unfolded states. Through influential studies of RNase A, Scheraga et al. proposed that oxidative folding is governed primarily by thiol-group accessibility, proximity, and reactivity (15). Thus, ordered tertiary structure can inhibit both disulfide-bond formation and reduction by burying oxidized cysteines or free thiolate groups, respectively. In this scheme formation of disulfide bridges may but need not precede formation of local structure (16).

The above perspective, supported by an extensive literature over the past four decades, is compatible with multiple oxidative routes to the native state as inferred from in vitro refolding studies of proinsulin (28). That similarity notwithstanding, the marked contrast in thermodynamic stabilities and conformational flexibility (or rigidity) between proinsulin and BPTI suggests that underlying biophysical principles differ in the two limiting cases: proteins of marginal stability with conformational plasticity (as exemplified by insulin) and proteins of extreme stability and internal rigidity (as exemplified by BPTI). The former class is likely to predominate in the vertebrate proteome.

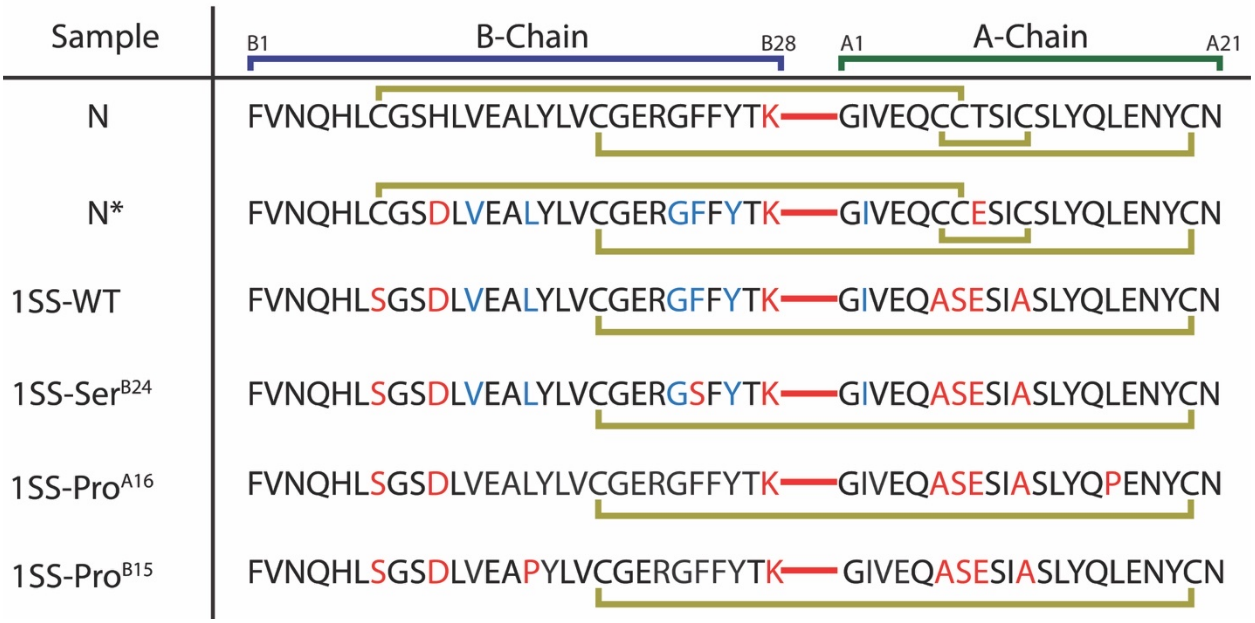


**SCHEME S1 |** Labeling design for variant DesDi protein sequences. Sample names at left report the presence of either one ([A20-B19]) or three (N and N*) disulfide bonds; yellow lines show disulfide linkages. A red line connecting the C-terminal B-domain and N-terminal A-domain sequences signifies the presence of a peptide bond between residues B28 and A1. All 1SS samples and N* have additional mutations $Glu^{A8}$ and $Asp^{B10}$ to augment segmental $\alpha$-helical propensity and to enhance solubility at neutral pH and the high protein concentrations needed for NMR spectroscopy. Residues in *red* highlight mutations to the WT insulin sequence whereas residues in *blue* are sites of $^{13}C$ isotopic labeling. N*, 1SS-WT, and 1SS-$Ser^{B24}$ were studied both as unlabeled and selectively $^{13}C$-labeled peptides.

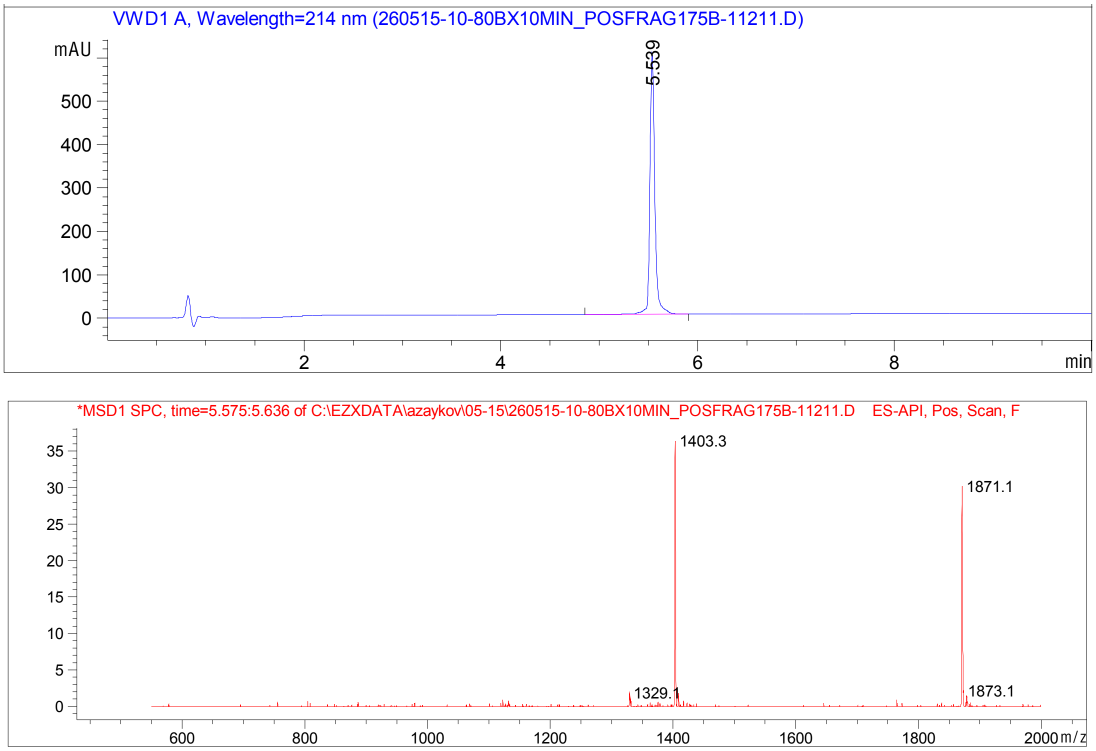


**FIGURE S1 |** LC-MS analysis of native-state $^{13}C$,$^{15}N$- [Glu$^{A8}$, Asp$^{B10}$] DesDi (N*). HPLC chromatogram monitored by UV absorbance at 215 nm is shown at the top panel. ESI-MS spectra are shown in bottom panel.

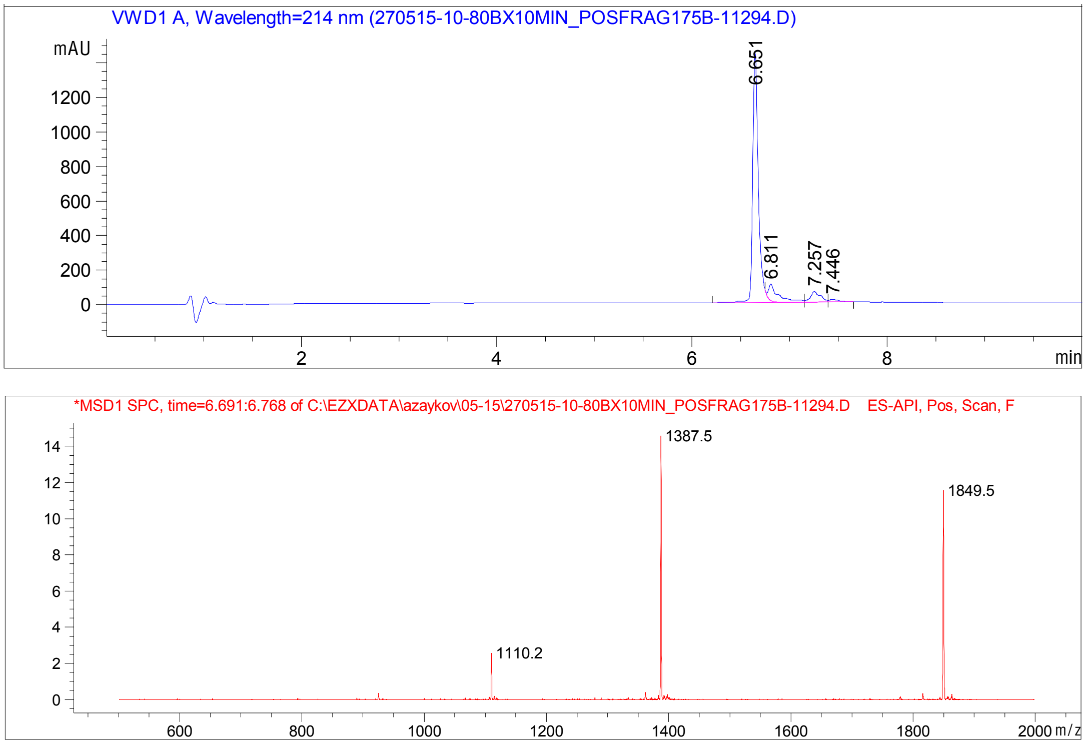


**FIGURE S2 |** LC-MS analysis of $^{13}C$,$^{15}N$-1SS DesDi. HPLC chromatogram monitored by UV absorbance at 215 nm is shown at the top panel. ESI-MS spectra are shown in bottom panel.

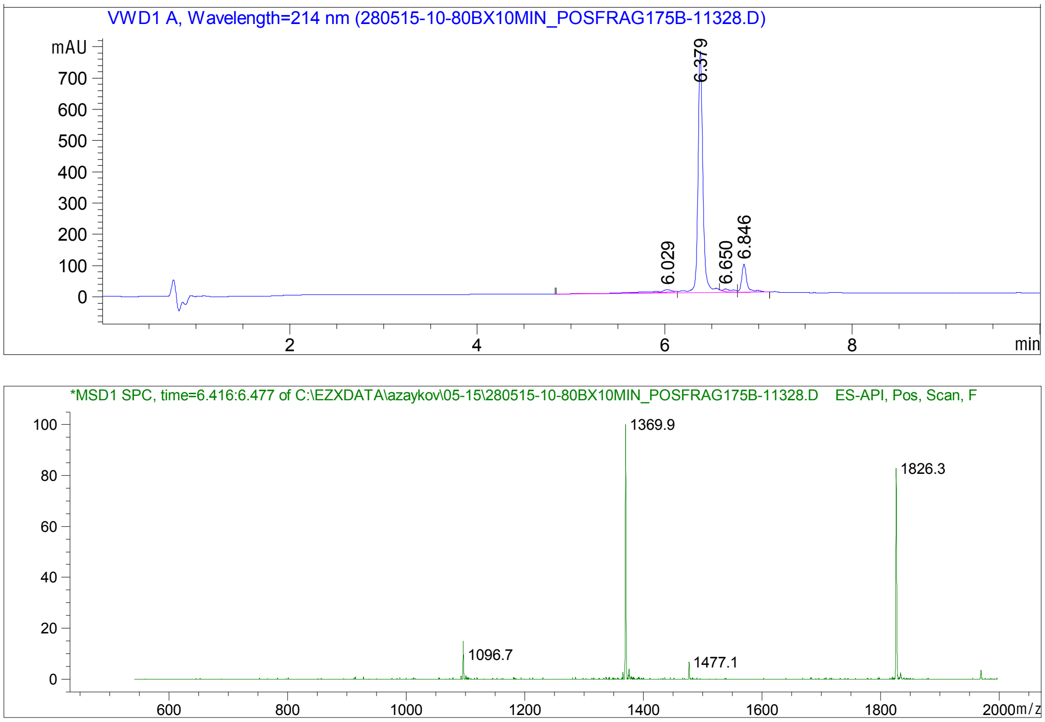


**FIGURE S3 |** LC-MS analysis of $^{13}C$,$^{15}N$-1SS- Ser$^{B24}$ DesDi. HPLC chromatogram monitored by UV absorbance at 215 nm is shown at the top panel. ESI-MS spectra are shown in bottom panel.

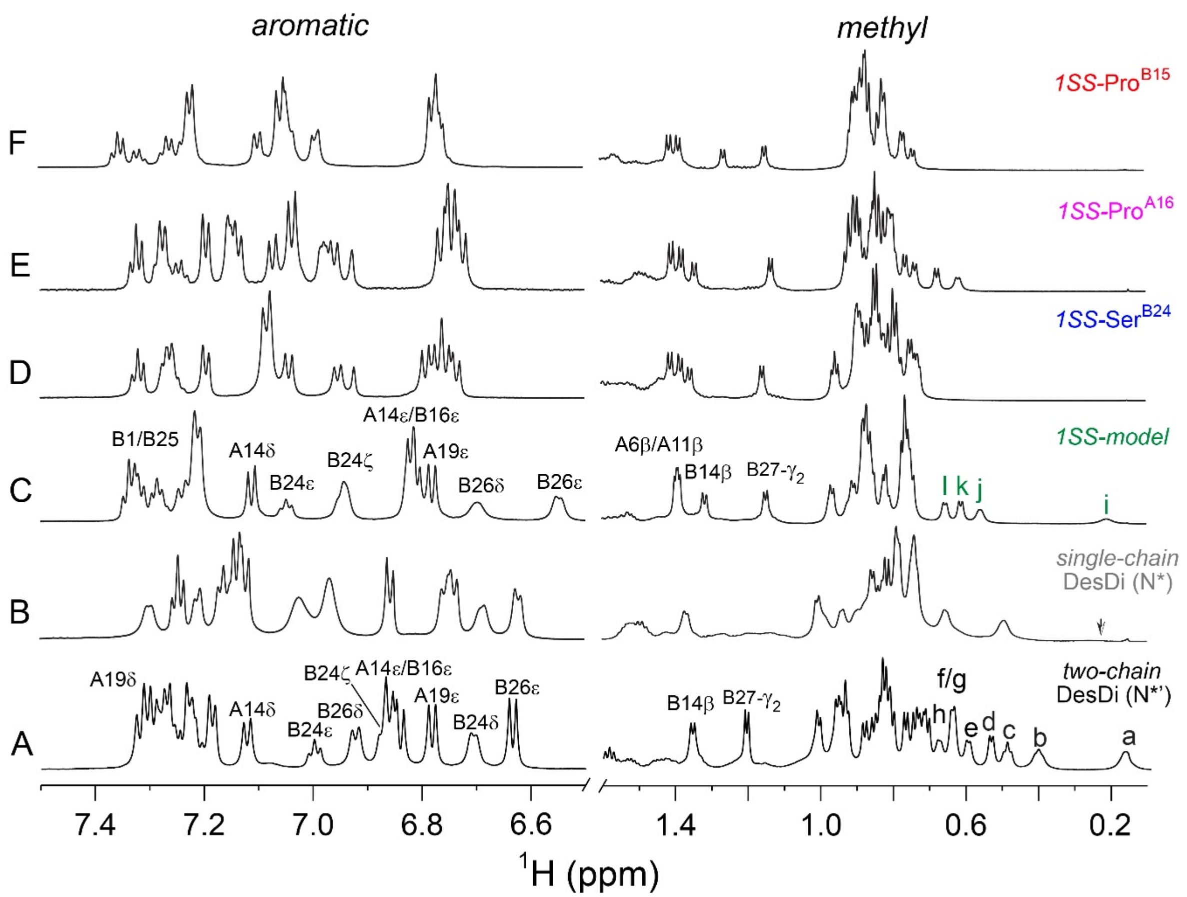


**FIGURE S4 |** Stack plot of 1D $^{1}$H-NMR spectra of DesDi insulin analogs: aromatic (*left panel*) and methyl (*right panel*) region. **(A)** two-chain DesDi (N*'). Selected resonance assignments are as indicated in the top of signals. The well-resolved methyl resonances are indicated as a: Leu$^{B15}$ $\delta_1$-$CH_3$; b: Ile$^{A2}$ $\delta_1$-$CH_3$; c: Ile$^{A10}$ $\delta_1$-$CH_3$; d: Leu$^{B15}$ $\delta_2$-$CH_3$; e: Ile$^{A2}$ $\gamma_2$-$CH_3$; f/g: Ile$^{A2}$ $\gamma_2$-$CH_3$ and Leu$^{B11}$ $\delta_1$-$CH_3$; h: Leu$^{B11}$ $\delta_2$-$CH_3$; **(B)** single-chain DesDi (N*). The arrow indicates broadening signal of Leu$^{B15}$ $\delta_1$-$CH_3$. **(C)** single-chain DesDi model with one disulfide bond at [A20-B19]. Cys$^{A6}$ and Cys$^{A11}$ were replaced by alanine, and Cys$^{A7}$ and Cys$^{B7}$ were mutated by serine. The 1SS model exhibits a spectral property of native-like insulin as observed in the aromatic region and in the upfield-shifted methyl region (*far right*). Selected resonance assignments are as indicated in the top of signals. The well-resolved methyl resonances are indicated as i: Leu$^{B15}$ $\delta_1$-$CH_3$; j: Leu$^{B11}$ $\delta_1$-$CH_3$; k: Leu$^{B15}$ $\delta_2$-$CH_3$ and l: Leu$^{B11}$ $\delta_2$-$CH_3$. **(D)** single-chain 1SS-Ser$^{B24}$ analog. **(E)** single-chain 1SS-Pro$^{A16}$ analog and **(F)** single-chain 1SS-Pro$^{B15}$ analog. Aromatic and methyl resonances of signature residues for three 1SS variants shifted to downfield, as well as exhibited a reduction in chemical-shift dispersion. Spectra were acquired at pD 7.4 (direct meter reading) at 35 °C in $D_2O$.

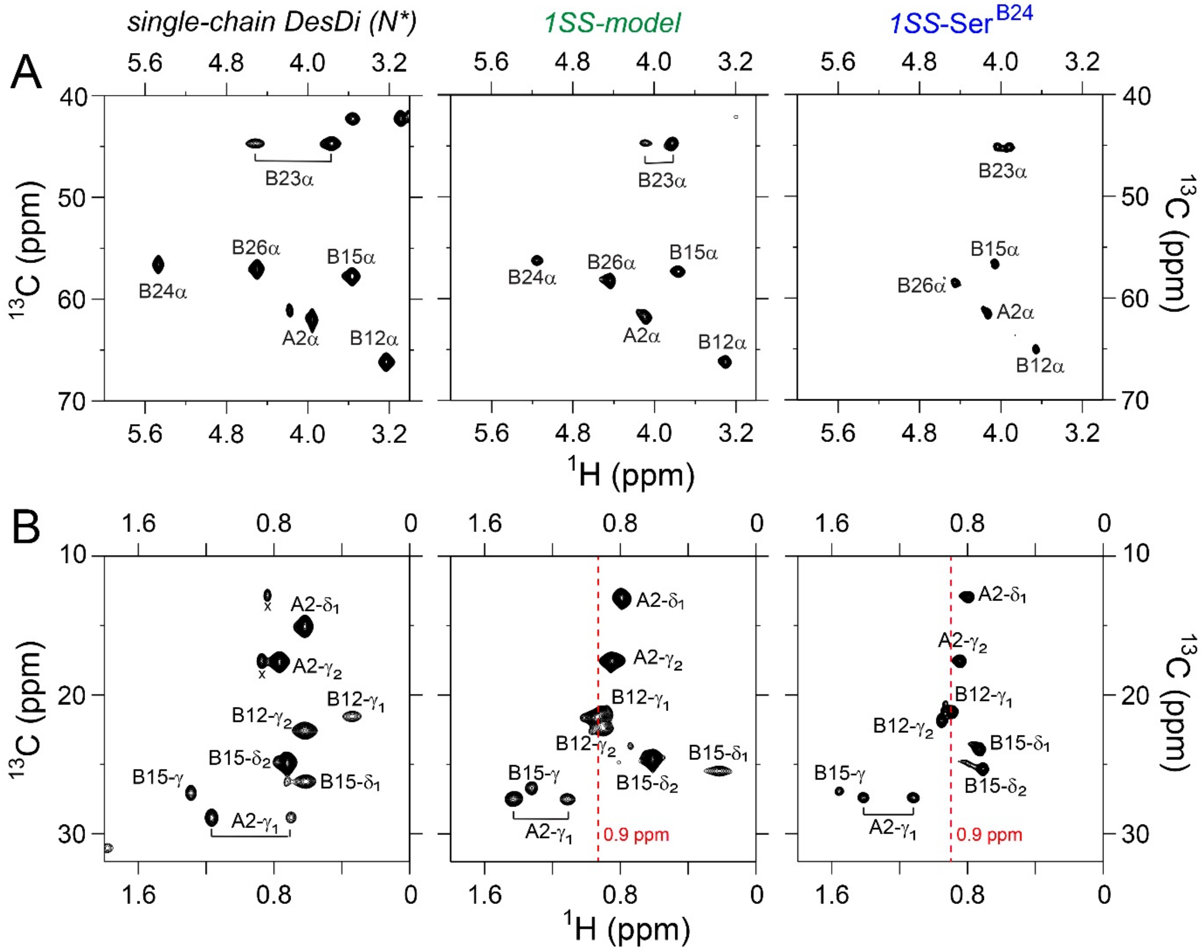


**FIGURE S5 |** $^{13}$C-HSQC of $^{13}$C-labeled DesDi analogs. Backbone fingerprint **(A)** and methyl carbon **(B)** regions of the $^{13}$C-HSQC spectra of selectively $^{13}$C-labeled DesDi analogs with resonance assignments determined from 3D HCC-TOCSY spectra (not shown). Red dashed line indicates the random coil shift of methyl resonance (~0.9 ppm).

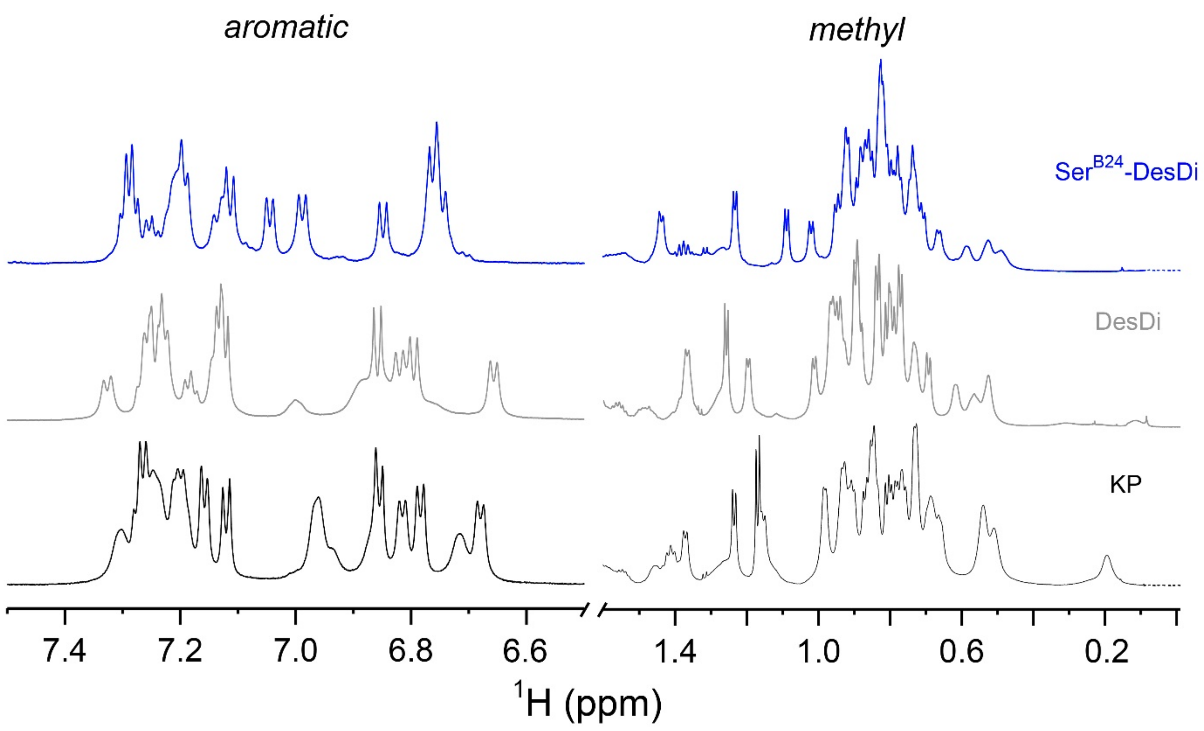


**FIGURE S6 |** Stack plot of aromatic and methyl regions of 1D $^{1}$H-NMR spectra of KP insulin (*blck; bottom panel*); DesDi insulin (*gray; middle panel*) and Ser$^{B24}$-DesDi analog (*blue; top panel*). Peaks found within -0.1 ppm ~ 0.08 ppm in the top and bottom panels were determined to be artifact based on the absence of corresponding cross-peaks in the 2D spectra; these regions were replaced with a dashed line. Spectra were acquired at pD 7.4 (direct meter reading) and at 35 °C in $D_2O$.

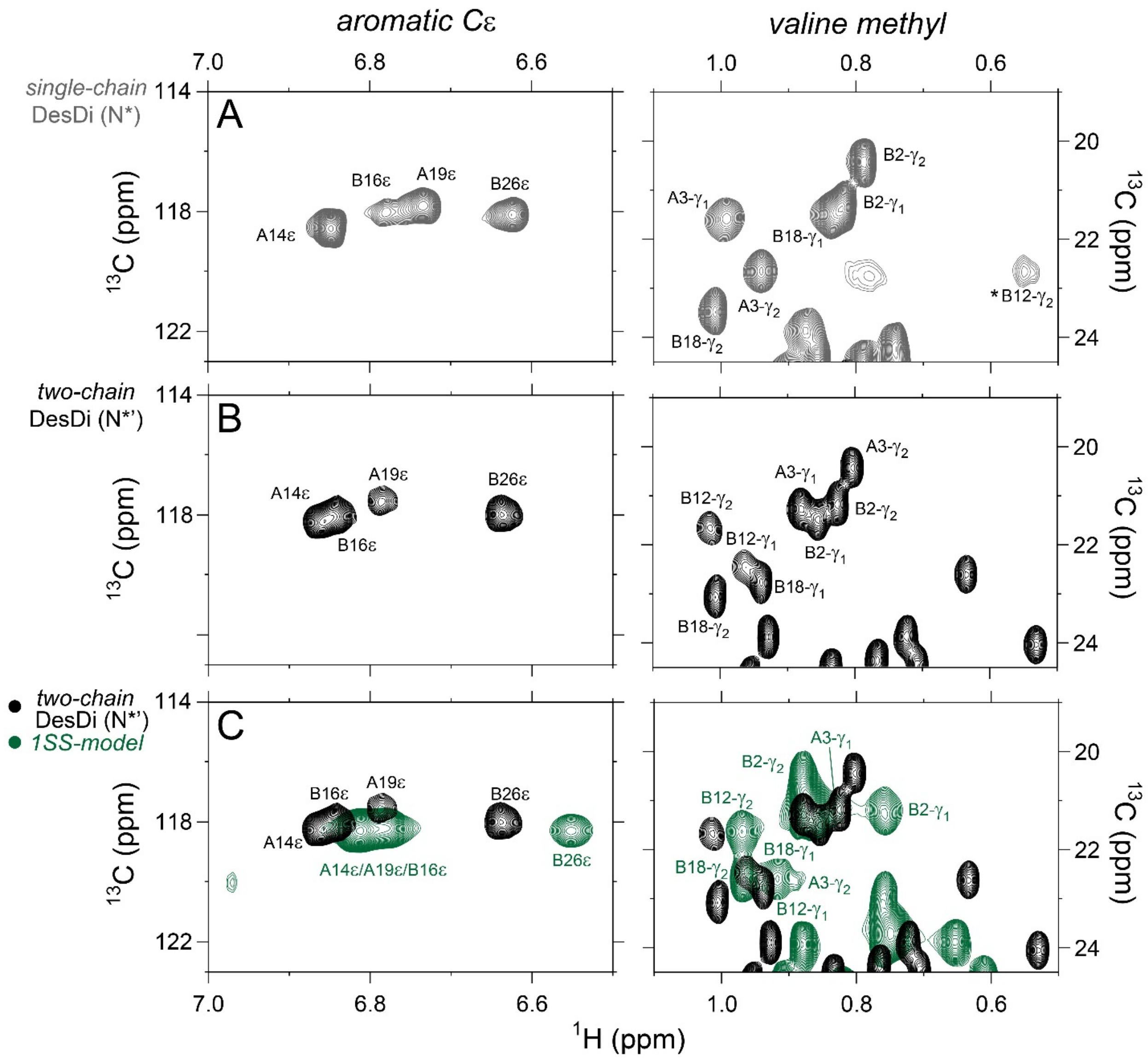


**FIGURE S7 |** Nature abundance $^{1}H$-$^{13}C$ HSQC spectra of single-chain DesDi analogs in aromatic Cε (*left panel*) and valine methyl region (*right panel*). **(A)** $^{1}H$-$^{13}C$ HSQC spectra of single-chain DesDi (N*, *gray*); **(B)** $^{1}H$-$^{13}C$ HSQC spectra of two-chain DesDi (N*', *black*); **(C)** spectral overlay of two-chain DesDi (N*', *black*) and single-chain DesDi *1SS*-model (*green*). Spectra were acquired at a $^{1}H$ frequency of 700 MHz at pD 7.4 (direct meter reading) and at 35 °C. Selected resonance assignments are as indicated.

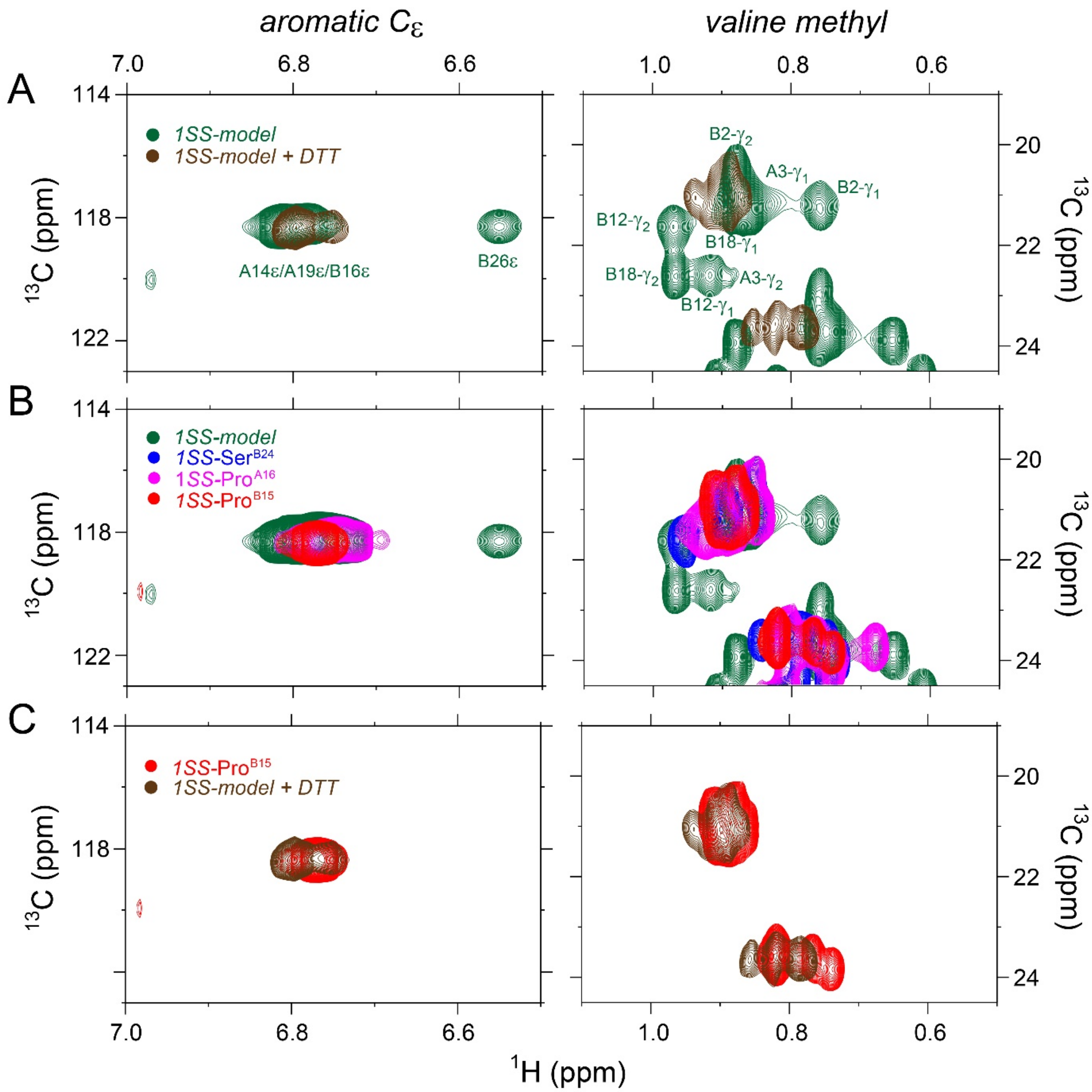


**FIGURE S8 |** Nature abundance $^{1}$H-$^{13}$C HSQC spectra of single-chain DesDi analogs in aromatic Cε (*left panel*) and valine methyl region (*right panel*). **(A)** spectral overlay of 1SS model (*green*) and a spectra after addition of 70 mM deuterated dithiothreitol (DTT) (*brown*); **(B)** spectral overlay of single-chain DesDi 1SS-model (*green*), 1SS-Ser$^{B24}$ variant (*blue*), single-chain 1SS-Pro$^{A16}$ variant (*magenta*) and single-chain 1SS-Pro$^{B15}$ variant (*red*); **(C)** spectral overlay of 1SS-Pro$^{B15}$ variant (*red*) and parent 1SS model after adding 70 mM deuterated dithiothreitol (*brown*). Spectra were acquired at a $^{1}$H frequency of 700 MHz at pD 7.4 (direct meter reading) and 35 °C. Selected resonance assignments are as indicated.

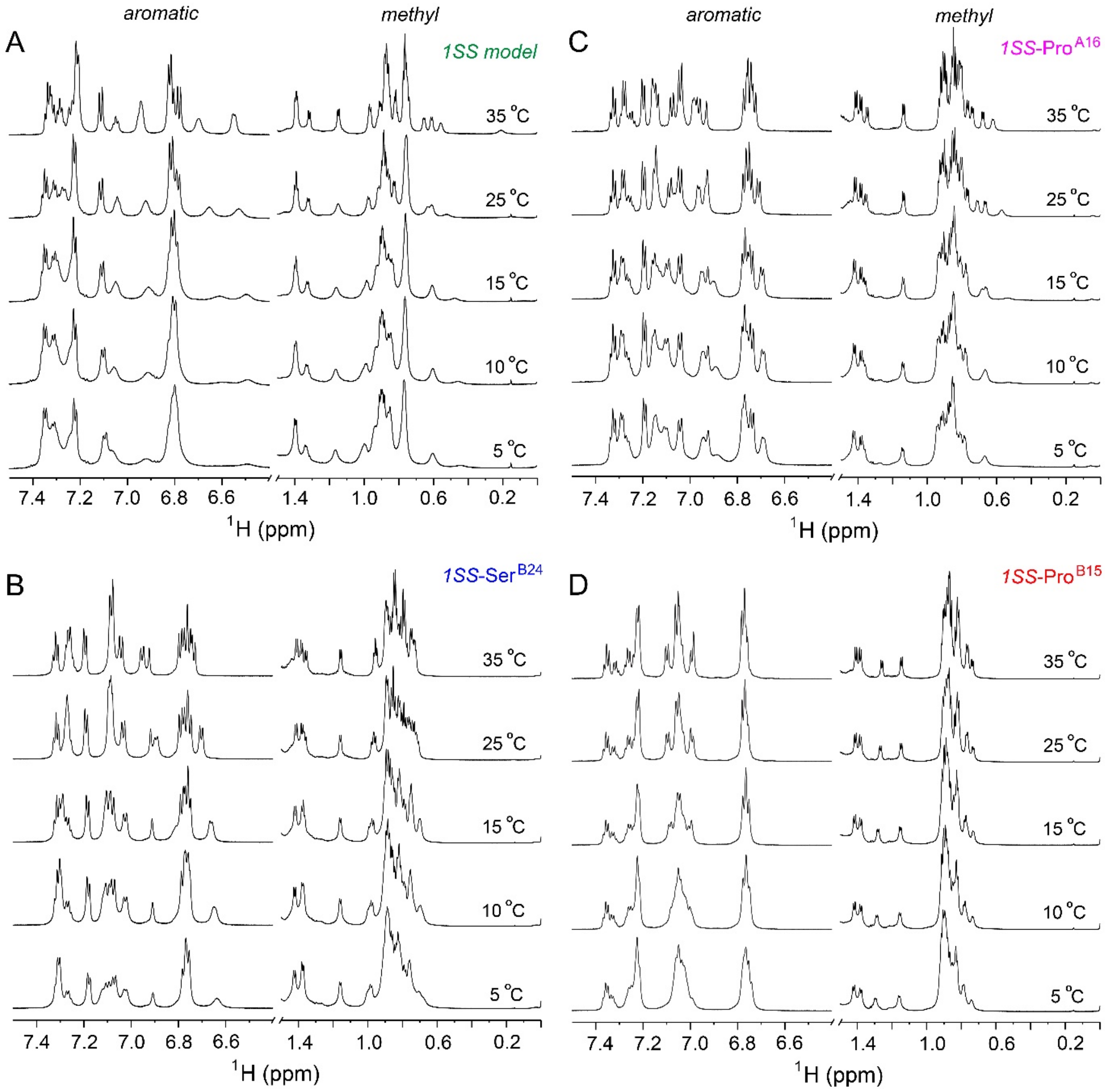


**FIGURE S9 |** Temperature-dependent $^1$H-NMR spectra in aromatic and methyl regions at 5, 10, 25, 15 and 35 °C (*from bottom to top*). **(A)** parent 1SS model; **(B)** 1SS-Ser$^{B24}$; **(C)** 1SS-Pro$^{A16}$ and **(D)** 1SS-Pro$^{B15}$ analog. Spectra were acquired at a $^1$H frequency of 700 MHz at pD 7.4 (direct meter reading) in $D_2O$.

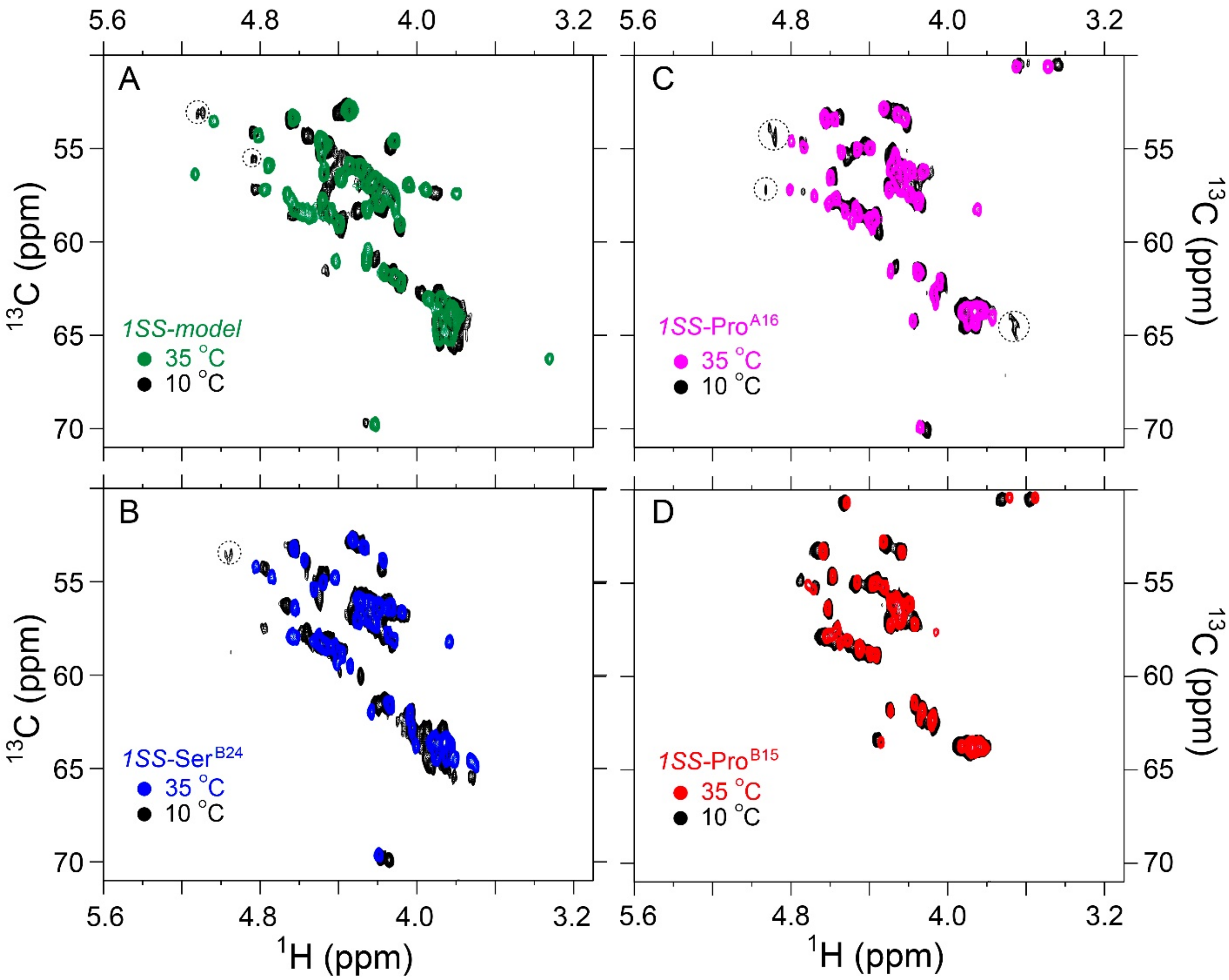


**FIGURE S10 |** $^{1}$H-$^{13}$C HSQC spectra of single-chain DesDi analogs in $^{1}$H$\alpha$/$^{13}$C$\alpha$ regions. **(A)** Spectral overlay of $^{1}$H-$^{13}$C HSQC of parent 1SS model at 35 °C (*green*) and 10 °C (*black*); **(B)** spectral overlay of $^{1}$H-$^{13}$C HSQC of 1SS-Ser$^{B24}$ at 35 °C (*blue*) and 10 °C (*black*); **(C)** spectral overlay of $^{1}$H-$^{13}$C HSQC of 1SS-Pro$^{A16}$ at 35 °C (*magenta*) and 10 °C (*black*); **(D)** spectral overlay of 1SS-Pro$^{B15}$ at 35 °C (*red*) and that at 10 °C (*black*). Due to linewidth exchange broadening, the peaks circled by dished line only displayed in the spectrum after intensity increasing 5-10 times. The $^{1}$H-NMR chemical shifts of those peaks were also confirmed in 2D NOESY or 2D TOCSY spectra. Spectra were acquired at a $^{1}$H frequency of 700 MHz at pD 7.4 (direct meter reading) and at 35 °C or at 10 °C.

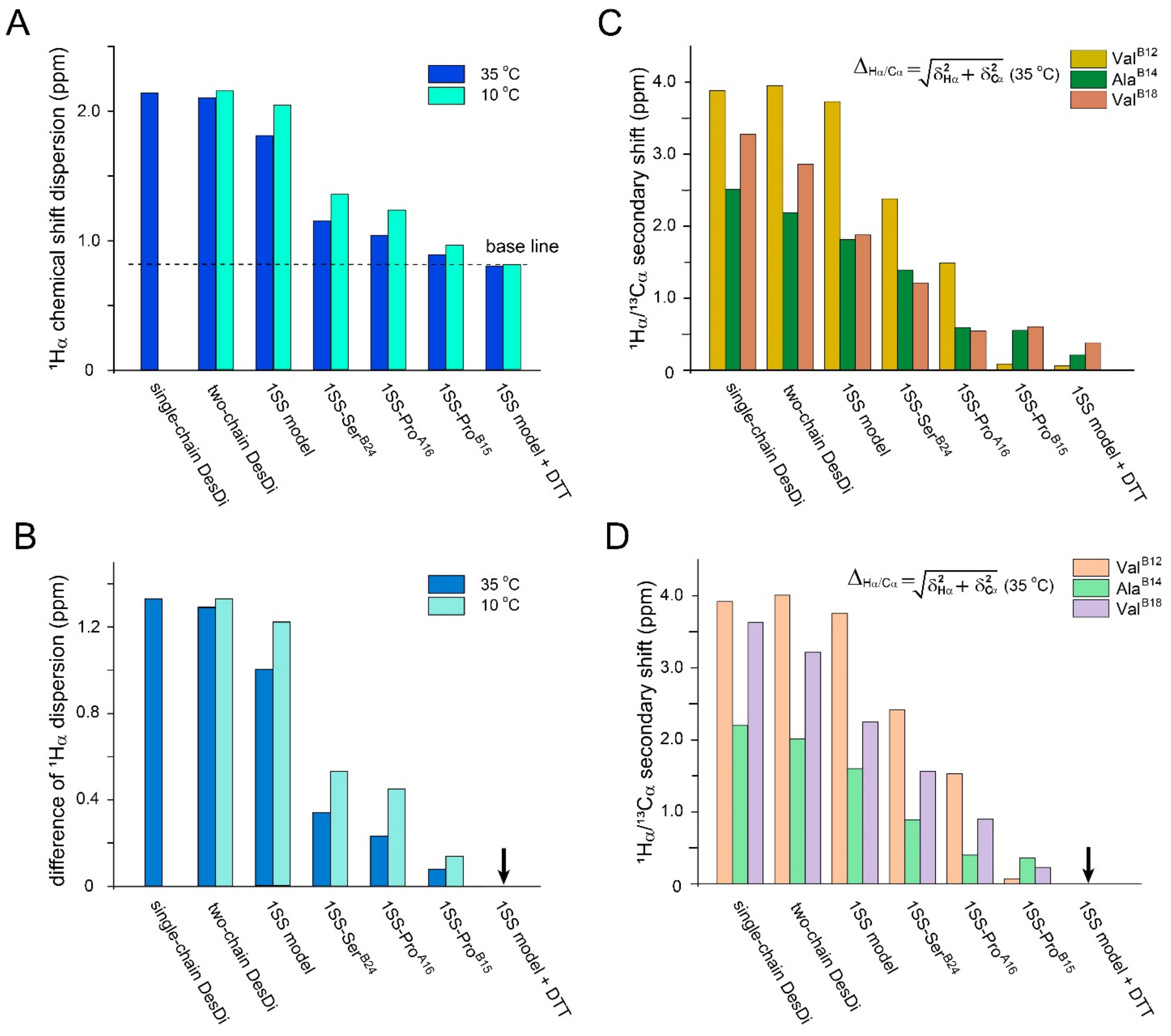


**FIGURE S11 |** The $^1H_\alpha$ chemical shift dispersion and $^1H_\alpha/^{13}C$ secondary chemical shift. **(A)** The $^1H_\alpha$ chemical shift dispersion of DesDi analogs at 35 and 10 °C, which was defined by $^1H_\alpha$ chemical shift range between minimum and maximum $^1H_\alpha$ chemical shift from 2D NMR spectra. The dotted line across panel A at the level of 1SS model + dithiothreitol (DTT) indicates schematically the new zero baseline in panel B. **(B)** The difference of $^1H_\alpha$ chemical shift dispersion using the level of 1SS model in the presence of dithiothreitol as zero baseline. **(C)** The $^1H_\alpha/^{13}C_\alpha$ secondary chemical shift for helix-related residues at 35 °C, which was calculated using equation $\Delta_{H\alpha/C\alpha} = \sqrt{\delta^2_{H\alpha} + \delta^2_{C\alpha}}$ $\delta_H$ and $\delta_C$ are the $^1H_\alpha$ and $^{13}C_\alpha$ chemical-shift difference between DesDi variants and random-coil shifts (29). **(D)** $^1H_\alpha/^{13}C_\alpha$ secondary shifts at 35 °C using the $^1H_\alpha/^{13}C_\alpha$ chemical shift of 1SS model in the presence of dithiothreitol as reference (secondary shifts are defined as difference between observed chemical shifts and tabulated random-coil values).

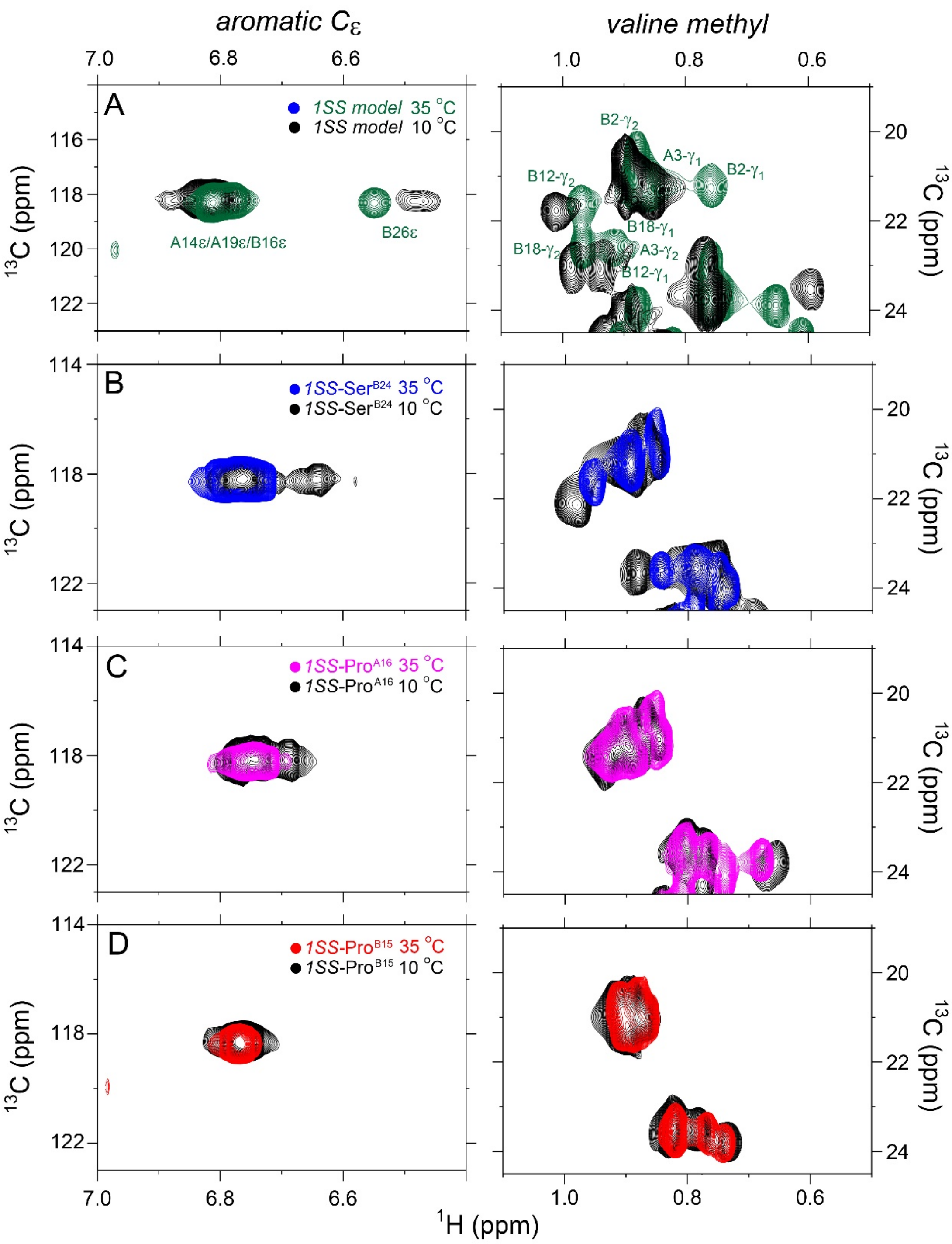


**FIGURE S12 |** Nature abundance $^{1}H$-$^{13}C$ HSQC spectra of single-chain DesDi analogs in aromatic Cε (*left panel*) and valine methyl region (*right panel*). **(A)** Spectral overlay of parent 1SS model at 35 °C (*blue*) and 10 °C (*black*); **(B)** spectral overlay of 1SS-Ser$^{B24}$ at 35 °C (*green*) and 10 °C (*black*); **(C)** spectral overlay 1SS-Pro$^{A16}$ at 35 °C (*magenta*) and 10 °C (*black*); **(D)** spectral overlay of 1SS-Pro$^{B15}$ at 35 °C (*red*) and 10 °C (*black*). Spectra were acquired at a $^{1}H$ frequency of 700 MHz at pD 7.4 (direct meter reading) in $D_2O$.

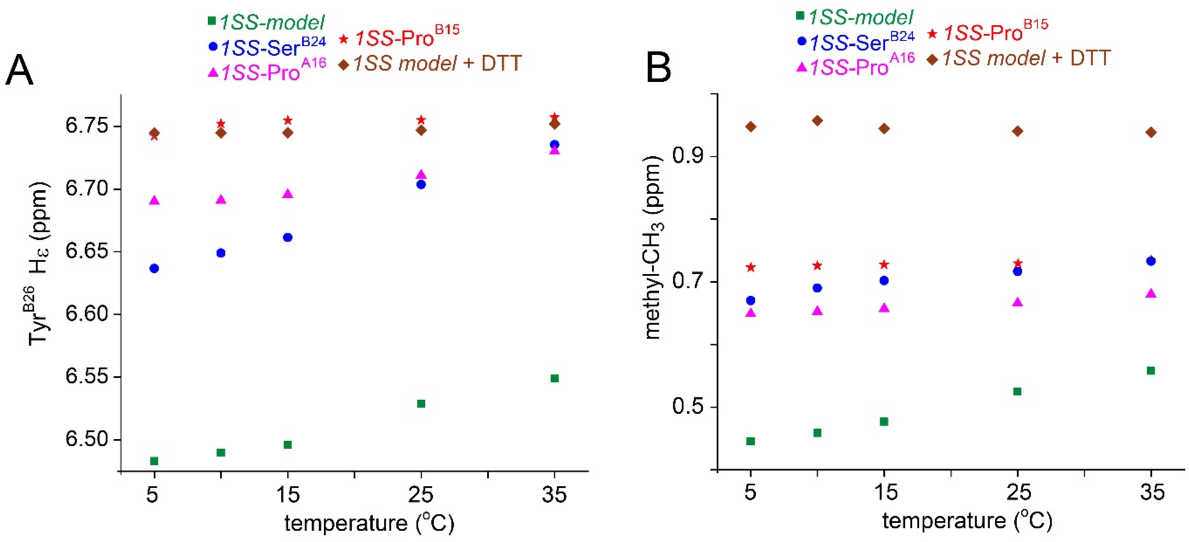


**FIGURE S13 |** Temperature-dependence of proton chemical shift. **(A)** The $^{1}$H-chemical shift temperature-dependence of Tyr$^{B26}$-Hε at 5, 10, 15, 25 and 35 °C. Parent 1SS model (*green square*); 1SS-Ser$^{B24}$ (*blue circle*); single-chain 1SS-Pro$^{A16}$ analog (*magenta triangle*); single-chain 1SS-Pro$^{B15}$ (*red star*) and control (*brown diamond,* reduced 1SS model in 70 mM dithiothreitol (DTT)). **(B)** The $^{1}$H-NMR chemical-shift temperature-dependence of a well-resolved methyl proton at 5, 10, 15, 25 and 35 °C. Color code is the same as in panel *A*.

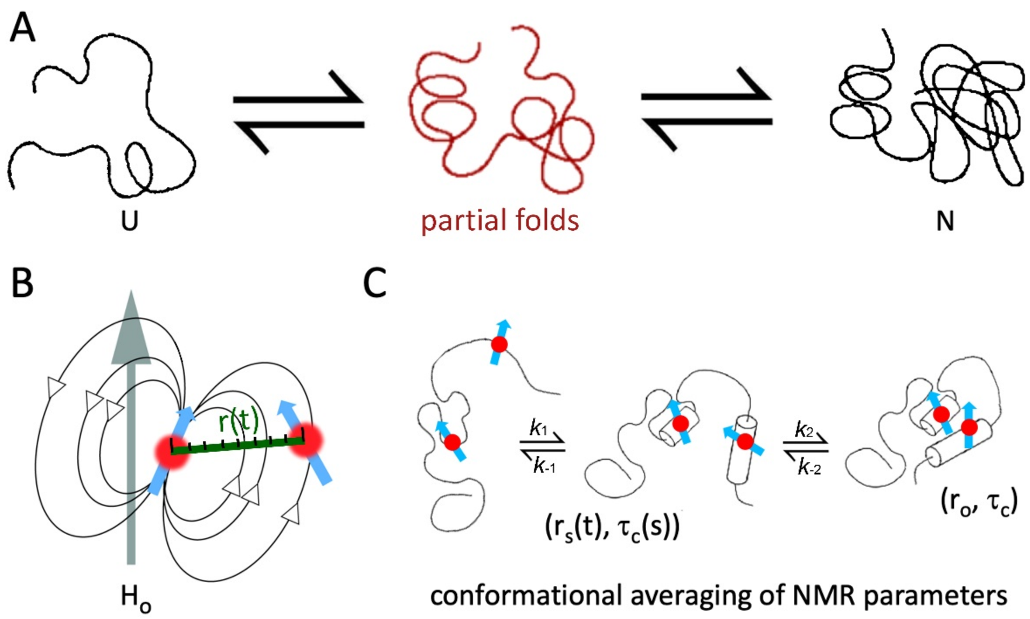


**FIGURE S14 |** General events during protein folding process and corresponding NMR averaging mechanisms. **(A)** Schematic representation of a general physical states of a polypeptide chain: (*left*) unstructured protein state or random coil, (*center*) partially folded intermediates (including molten globules) and (*right*) ordered native forms. **(B)** Magnetic dipole-dipole interactions underlying spin relaxation and nuclear Overhauser enhancements (NOEs). The *red* spheres denote spin-½ nuclei; *blue* arrows indicate their magnetic moments in the magnetic field (*gray*); the induced field lines of the left nucleus are shown in *black* and the internuclear distance in *green*. **(C)** Conformational averaging of NMR parameters (such as chemical shift, line width and NOE) due to exchange between different conformational states. Such conformational exchange can occur between overall states or as a result of local order/disorder transitions.

NMR spectroscopy enables dynamic features of a protein ensemble to be probed (30, 31). Parameters include chemical shift, J-coupling constant, NOEs, residual decoupling constant (RDC) and relaxation times; these can provide insight into many aspects of protein folding.

(i) Degree of $^{1}H$ and $^{15}N$ chemical-shift dispersion (32) informs extent of organization; in particular, secondary structure may be inferred from patterns of specific $^{1}H$, $^{13}C$ and $^{15}N$ chemical shifts (32) as in the present study (**Figure 4** in main text).

(ii) Tertiary structure may be modeled by restrained molecular dynamics based on structural assumptions or based on observed NMR parameter-derived structure restraints, such as NOE-derived distance restraints and chemical-shift or J-derived dihedral-angle restraints (33) (**Figure 9A** in main text).

(iii) $^{15}N$ and $^{13}C$ relaxation times may also be used to characterize the dynamics of protein folding intermediates (34, 35) (not measured in the present study).

NMR parameters can be influenced by exchange processes that are reversible and characterized as *fast*, *intermediate* or *slow* on the time scale of NMR chemical shifts. In the case of slow exchange (milliseconds or longer; $k_{ex} >> |\Delta\omega_i|$), separate signal components

are resolved (36); distinct chemical shifts and other NMR parameters can be extracted (26). In intermediate exchange NMR signals are typically too broad to observe (37). If exchange is fast ($k_{ex} << |\Delta\omega_i|$), the different signal components are not resolved: conformational averaging of NMR parameters depends on the parameter. The simplest case is chemical shift, which will be linearly weighted according to subpopulations (for example, $<\delta_{ave}> = f_U\delta_U + f_I\delta_I + f_N\delta_N$ where $f_U + f_I + f_N = 1$; where subscripts U, I and N refer to the unfolded, intermediate and native states, respectively). In the present study fast exchange leads to trends in dispersion among the 1SS peptides: wildtype > $Ser^{B24}$ > $Pro^{A16}$ > $Pro^{B15}$ as described in the main text. More complex is the averaging of inter-residue NOEs, which in favorable cases can be transferred between conformational states (exchange-transferred NOEs, trNOE); an ensemble that is predominantly unfolded can thus exhibit long-range NOEs "imprinted" by a transient globular state. We speculate that some long-range NOEs in the spectrum of the disordered $Pro^{B15}$ 1SS peptide arise by this mechanism.

Influential NMR studies of one-disulfide folding intermediates of BPTI in the 1990s provided important insights into the dynamics of protein-folding intermediates in the case of a highly stable protein (see Supplemental Discussion). These studies exploited observation of slow-exchange cross peaks (26), intermediate-exchange broadening (38), changes in rates of aromatic ring rotation ("ring flips") (39), changes in rates of amide-proton exchange in $D_2O$ (40) and $^{15}N$-$^{1}H$ relaxation times and heteronuclear NOEs (41).

**TABLE S1.** Molecular properties of peptide models and insulin analogs[a]

| sample Name | Retention Time (min) | Molar Mass (Da) Theoretical / Actual | $\Delta G_u$ (kcal/mol)[b] | CD % α -helix[e,f] | Fold ER stress activation (pPERK/PERK)[g] |
|---|---|---|---|---|---|
| Insulin *lispro* | 17.12 | 5807.5 / 5806.7 | 3.0 ± 0.1 | 38 – 40% | |
| Wild-type Insulin | 17.28 | 5807.5 / 5806.2 | 3.1 ± 0.1 | 47 – 56% | |
| N | 14.77 | 5591.4 / 5590.4 | >4[c] | 49 – 64% | |
| N* | 15.20 | 5597.3 / 5596.2 | 7.6 ± 0.4[c] | 49 – 58% | |
| 1SS-WT | 23.57 | 5505.0 / 5504.2 | 0.9 ± 0.2 | 27 – 30% | 1.00 ± 0.32 |
| 1SS-Ser$^{B24}$ | 22.03 | 5445.0 / 5444.0 | ND[d] | 19 – 20% | 0.98 ± 0.13 |
| 1SS-Pro$^{B15}$ | 18.77 | 5489.0 / 5488.4 | ND[d] | 10 – 11% | 4.18 ± 0.57 |
| 1SS-Pro$^{A16}$ | 21.71 | 5489.0 / 5488.1 | ND[d] | 12 – 14% | 9.65 ± 1.27 |

[a]The analytical LC-MS retention times and theoretical and experimentally measured molar masses are given for all DesDi species not labeled with $^{13}C$

[b]$\Delta G_u$ values provided were obtained from curve fitting of CD-guanidine titrations to a two-state unfolding transition model as described in methods.

[c]CD-guanidine titrations performed at 37°C for N and 50°C N* (see **Figure 4** in companion paper (24)) yielded partially folded peptides at the maximum the guanidine concentration. Fitting of the N* titration was successful ($R^2$=0.9994), but analysis of the N titration curve could only place a lower limit on thermodynamic stability of 4 kcal/mol.

[d]ND, not determined due to lack of a well-defined ellipticity baseline corresponding to the folded state.

[e]Total percent α-helix, β-sheet, and disordered coil were obtained from spectra acquired at discreet temperatures of 4°C, 25°C, and 37°C using the SELCON-3 algorithm (42-44). Estimated percentages are presented as the minimum to maximum content calculated across the three sampled temperatures.

[f]Estimates were calculated from WT insulin spectra to confirm that SELCON-3 processing of our own CD data gives values that match those published in the literature (45).

[g] The data represents ER stress levels in the full-length proinsulin framework.

**TABLE S2.** $^1$H chemical shifts for all labeled DesDi samples[a]

| | Residue | $H_\alpha$ | $H_\beta$ | Others |
|---|---|---|---|---|
| Single chain DesDi (N*) | B12 | 3.26 | 1.79 | $C\gamma_1H$ 0.62; $C\gamma_2H$ 0.34 |
| | B15 | 3.57 | 0.93 , -0.05 | $C\delta_1H$ 0.62; $C\delta_2H$ 0.72; $C\gamma H$ 1.29 |
| | B23 | 3.77, 4.52 | | |
| | B24 | 5.47 | 3.06, 3.56 | $C\delta H$ 6.95; $C\epsilon H$ 6.96; $C\zeta H$ 7.04 |
| | B26 | 4.50 | 2.23 | $C\delta H$ 6.71; $C\epsilon H$ 6.63 |
| | A2 | 3.96 | 1.85 | $C\delta H$ 0.62; $C\gamma_1H$ 1.17; 0.70; $C\gamma_2H$ 0.77 |
| 1SS model | B12 | 3.32 | 2.05 | $C\gamma_1H$ 0.95; $C\gamma_2H$ 0.90 |
| | B15 | 3.77 | 1.10 , 0.33 | $C\delta_1H$ 0.22; $C\delta_2H$ 0.61; $C\gamma H$ 1.32 |
| | B23 | 4.09, 3.82 | | |
| | B24 | 5.16 | 2.88 , 3.19 | $C\delta H$ 6.82; $C\epsilon H$ 6.94; $C\zeta H$ 7.05 |
| | B26 | 4.43 | 2.89 , 2.66 | $C\delta H$ 6.70; $C\epsilon H$ 6.55 |
| | A2 | 4.008 | 1.81 | $C\delta H$ 0.79; $C\gamma_1H$ 1.43 , 1.11; $C\gamma_2H$ 0.85 |
| 1SS-Ser$^{B24}$ [b] | B12 | 3.68 | 2.08 | $C\gamma_1H$ 0.89; $C\gamma_2H$ 0.95 |
| | B15 | 4.05 | 1.65, 1.34 | $C\delta_1H$ 0.71; $C\delta_2H$ 0.73; $C\gamma H$ 1.56 |
| | B23 | 4.03 , 3.94 | | |
| | B26 | 4.45 | 2.77 , 2.93 | $C\delta H$ 6.95; $C\epsilon H$ 6.73 |
| | A2 | 4.13 | 1.83 | $C\delta H$ 0.80; $C\gamma_1H$ 1.42, 1.12; $C\gamma_2H$ 0.85 |

[a]All chemical shifts were calibrated in parts per million (ppm) relative to 4,4-dimethyl-4-silapentane-1-sulfonic acid (DSS) as an internal standard, which was set to 0ppm.
[b]1SS-Ser$^{B24}$ has a serine residue at position B24 that is not $^{13}$C-labeled.

**TABLE S3.** $^{13}C$ chemical shifts for all labeled DesDi samples[a]

| | Residue | Cα | Cβ | Others |
|---|---|---|---|---|
| Single chain DesDi (N*) | B12 | 66.39 | 31.07 | $C\gamma_1$ 21.57; $C\gamma_2$ 22.59 |
| | B15 | 57.70 | 40.62 | $C\delta_1$ 26.35; $C\delta_2$ 24.95; Cγ 27.10 |
| | B23 | 44.66 | | |
| | B24 | 56.54 | 42.23 | Cδ 133.06; Cϵ 130.95; Cζ128.84 |
| | B26 | 57.06 | 39.74 | Cδ 132.77; Cϵ 118.05 |
| | A2 | 62.03 | 38.78 | Cδ 15.14; $C\gamma_1$ 28.89; $C\gamma_2$ 17.65 |
| 1SS model | B12 | 66.24 | 31.69 | $C\gamma_1$ 21.62; $C\gamma_2$ 21.46 |
| | B15 | 57.35 | 40.73 | $C\delta_1$ 25.53; $C\delta_2$ 24.68; Cγ 26.78 |
| | B23 | 44.70 | | |
| | B24 | 56.31 | 42.16 | Cδ 132.59; Cϵ 131.22; Cζ 130.08 |
| | B26 | 58.22 | 39.59 | Cδ 132.79; Cϵ 118.31 |
| | A2 | 61.74 | 38.35 | Cδ 13.04; $C\gamma_1$ 27.47; $C\gamma_2$ 17.55 |
| 1SS-Ser$^{B24b}$ | B12 | 64.94 | 32.05 | $C\gamma_1$ 21.25; $C\gamma_2$ 21.84 |
| | B15 | 56.60 | 41.91 | $C\delta_1$ 25.38; $C\delta_2$ 23.93; Cγ 26.94 |
| | B23 | 45.18 | | |
| | B26 | 58.56 | 39.04 | Cδ 133.04; Cϵ 118.20 |
| | A2 | 61.57 | 38.58 | Cδ 12.99; $C\gamma_1$ 27.42; $C\gamma_2$ 17.60 |

[a]All chemical shifts were calibrated in parts per million (ppm) relative to 4,4-dimethyl-4-silapentane-1-sulfonic acid (DSS) as an internal standard, which was set to 0ppm.
[b]1SS-Ser$^{B24}$ has a serine residue at position B24 that is not $^{13}C$-labeled.

**TABLE S4.** $^{1}$H/$^{13}$C chemical shifts of aromatic groups of DesDi analogs[a]. Data were acquired at $^{1}$H frequency of 700 MHz and at pD7.4 (direct meter reading) and at 35 ºC.

| residue | Tyr$^{A14}$ | | Tyr$^{A19}$ | | Phe$^{B1}$ | | | Tyr$^{B16}$ | |
|---|---|---|---|---|---|---|---|---|---|
| $^{1}$H/$^{13}$C | Hδ/Cδ | Hε/Cε | Hδ/Cδ | Hε/Cε | Hδ/Cδ | Hε/Cε | Hζ/Cζ | Hδ/Cδ | Hε/Cε |
| single-chain N* | 7.12/133.27 | 6.85/118.52 | 7.27/133.87 | 6.74/117.75 | 7.14/132.19 | 7.25/131.35 | 7.18/129.65 | 6.95/130.92 | 6.79/117.97 |
| two-chain N* | 7.12/133.19 | 6.86/118.24 | 7.32/133.86 | 6.78/117.54 | 7.19/131.94 | 7.29/131.96 | 7.24/129.96 | 7.28/133.19 | 6.84/118.02 |
| 1SS model | 7.11/133.15 | 6.82/118.31 | 7.24/133.21 | 6.78/118.22 | 7.22/132.16 | 7.34/131.73 | 7.30/130.31 | 7.21/133.27 | 6.81/118.31 |
| 1SS-Ser$^{B24}$ | 7.04/133.14 | 6.76/118.20 | 7.08/133.08 | 6.79/118.26 | 7.19/132.09 | 7.32/131.49 | 7.26/129.89 | 7.08/133.08 | 6.77/118.18 |
| 1SS-Pro$^{A16}$ | 7.04133.23 | 6.75/118.24 | 7.08/133.34 | 6.76/118.24 | 7.20/132.17 | 7.33/131.58 | 7.28/129.27 | 7.04133.23 | 6.75/118.24 |
| 1SS-Pro$^{B15}$ | 7.06/133.28 | 6.78/118.33 | 7.06/133.28 | 6.78/118.33 | 7.22/132.10 | 7.35/131.75 | 7.32/130.30 | 7.06/133.28 | 6.78/118.33 |
| 1SS model+DTT | 7.08/133.25 | 6.79/118.29 | 7.08/133.25 | 6.79/118.29 | 7.23/130.40 | 7.36/129.9 | 7.33/128.3 | 7.10/133.25 | 6.81/118.29 |

| residue | Phe$^{B24}$ | | | Phe$^{B25}$ | | | Tyr$^{B26}$ | |
|---|---|---|---|---|---|---|---|---|
| $^{1}$H/$^{13}$C | Hδ/Cδ | Hε/Cε | Hζ/Cζ | Hδ/Cδ | Hε/Cε | Hζ/Cζ | Hδ/Cδ | Hε/Cε |
| single-chain N* | 6.95/133.06 | 7.04/130.95 | 7.04/128.84 | 7.12/133.27 | 7.23/132.24 | 7.18/129.65 | 6.71/132.77 | 6.63/118.05 |
| two-chain N* | 6.70/132.76 | 6.87/130.99 | 6.99/130.19 | 7.23/132.16 | 7.26/131.16 | 7.22/130.22 | 6.92/132.69 | 6.64/117.97 |
| 1SS model | 6.82/132.59 | 6.94/131.22 | 7.05/130.08 | 7.22/132.16 | 7.32/131.61 | 7.28/130.07 | 6.70/132.79 | 6.55/118.31 |
| 1SS-Ser$^{B24}$ | N/A | N/A | N/A | 7.08/131.96 | 7.26/131.46 | 7.25/129.95 | 6.95/133.04 | 6.73/118.20 |
| 1SS-Pro$^{A16}$ | 6.99/132.16 | 7.16/131.42 | 7.16/129.95 | 7.14/132.05 | 7.28/131.48 | 7.25/129.99 | 6.96/133.20 | 6.73/118.24 |
| 1SS-Pro$^{B15}$ | 7.04/131.91 | 7.22/131.61 | 7.22/129.90 | 7.10/132.00 | 7.26/131.45 | 7.24/129.95 | 6.99/133.33 | 6.76/118.33 |
| 1SS model+DTT | 7.07/131.86 | 7.25/131.42 | 7.23/129.82 | 7.11/131.96 | 7.26/131.42 | 7.25/129.92 | 7.03/133.20 | 6.76/118.24 |

[a]All chemical shifts were calibrated in parts per million (ppm) relative to 4,4-dimethyl-4-silapentane-1-sulfonic acid (DSS) as an internal standard, which was set to 0 ppm. DTT-dithiothreitol

**TABLE S5**. The $^1H_\alpha/^{13}C_\alpha$ chemical shift and $^1H_\alpha/^{13}C_\alpha$ secondary shift of selected residues in DesDi analogs at 35 °C [a]

| | $Val^{B12}$ | | $Ala^{B14}$ | | $Val^{B18}$ | | average |
|---|---|---|---|---|---|---|---|
| | $^1H_\alpha/^{13}C_\alpha$ | Δ1/Δ2[b] | $^1H_\alpha/^{13}C_\alpha$ | Δ1/Δ2[b] | $^1H_\alpha/^{13}C_\alpha$ | Δ1/Δ2[b] | $\overline{\Delta2}$[c] |
| single-chain N* | 3.26/66.39 | 3.88/3.92 | 4.04/55.31 | 2.51/2.20 | 3.69/65.85 | 3.27/3.63 | 3.33 |
| two-chain N* | 3.20/66.47 | 3.97/4.01 | 4.07/55.00 | 2.20/2.01 | 3.76/65.45 | 2.87/3.22 | 3.19 |
| 1SS model | 3.32/66.24 | 3.73/3.76 | 4.11/54.60 | 1.80/1.60 | 3.86/64.48 | 1.89/2.25 | 2.69 |
| 1SS-$Ser^{B24}$ | 3.68/64.94 | 2.38/2.42 | 4.17/53.89 | 1.39/0.89 | 4.00/63.81 | 1.23/1.56 | 1.74 |
| 1SS-$Pro^{A16}$ | 3.77/64.05 | 1.49/1.53 | 4.22/53.40 | 0.59/0.40 | 4.06/63.14 | 0.54/0.90 | 1.05 |
| 1SS-$Pro^{B15}$ | 4.08/62.62 | 0.08/0.07 | 4.23/53.36[d] | 0.55/0.36 | 4.08/62.01 | 0.60/0.23 | 0.25 |
| 1SS model+DTT | 4.11/62.56 | 0.05/0 | 4.22/53.00 | 0.21/0 | 4.08/62.24 | 0.38/0 | / |
| random coil[e] | 4.16/62.61 | / | 4.35/52.82 | / | 4.16/62.61 | / | / |

[a]All chemical shifts were calibrated in parts per million (ppm) relative to 4,4-dimethyl-4-silapentane-1-sulfonic acid (DSS) as an internal standard, which was set to 0 ppm.

[b]The $^1H_\alpha/^{13}C_\alpha$ secondary chemical shift was calculated according the equation $\Delta_{H\alpha/C\alpha} = \sqrt{\delta_{H\alpha}^2 + \delta_{C\alpha}^2}$. $\delta_{H\alpha}$ and $\delta_{C\alpha}$ are the $^1H_\alpha$ and $^{13}C_\alpha$ secondary chemical shift and are the differences between observed chemical shift of DesDi variants and random coil shifts from literature (Δ1), or the shifts of random coil base line of single-chain DesDi 1SS-model in 70 mM deuterated dithiothreitol (DTT) (Δ2).

[c]Average $^1H_\alpha/^{13}C_\alpha$ secondary shift ($\bar{\Delta}$) of three α-helix related residues ($Val^{B12}$, $Ala^{B15}$ and $Val^{B18}$) was calculated using equation $\bar{\Delta} = \sqrt{(\Delta_{B12}^2 + \Delta_{B14}^2 + \Delta_{B18}^2)/3}$. $\Delta_{B12}^2$, $\Delta_{B14}^2$ and $\Delta_{B18}^2$ are $^1H_\alpha/^{13}C_\alpha$ secondary shift of residues $Val^{B12}$, $Ala^{B15}$ and $Val^{B18}$, respectively. $\overline{\Delta2}$ is the average secondary shift random coil shift base line of single-chain DesDi 1SS-model in 70 mM deuterated dithiothreitol.

[d]The $^{13}C_\alpha$ resonance of $Ala^{B14}$ shifted toward upfield due to proline replacement at B15 position; The secondary shift of alanine in 1SS-$Pro^{B15}$ was calculated using $Ala^{A11}$.

[e]The $^1H_\alpha/^{13}C_\alpha$ chemical shifts of random coil were measured in aqueous solutions of the linear pentapeptide (GGXGG) (29).

**TABLE S6**. The $^1H_\alpha/^{13}C_\alpha$ chemical shift of selected residues in DesDi analogs at 10 °C[a]

| | Val[B12] | | Ala[B14] | | Val[B18] | |
|---|---|---|---|---|---|---|
| | $^1H_\alpha/^{13}C_\alpha$ | Δ1/Δ2[b] | $^1H_\alpha/^{13}C_\alpha$ | Δ1/Δ2[b] | $^1H_\alpha/^{13}C_\alpha$ | Δ1/Δ2[b] |
| two-chain | 3.21/66.84 | 4.33/4.37 | 4.13/55.24 | 2.43/2.24 | 3.76/65.71 | 3.12/3.48 |
| 1SS model | 3.21/66.90 | 4.39/4.43 | 4.13/54.89 | 2.08/1.89 | 3.80/65.44 | 2.85/3.21 |
| 1SS-Ser[B24] | 3.63/65.96 | 3.39/3.43 | 4.17/54.32 | 1.51/1.32 | 3.96/64.42 | 1.82/2.18 |
| 1SS-Pro[A16] | 3.65/64.45 | 1.90/1.85 | 4.21/53.63 | 0.82/0.63 | 4.01/63.40 | 0.95/1.16 |
| 1SS-Pro[B15] | 4.08/62.38 | 0.24/0.18 | 4.23/53.23[c] | 0.43/0.23 | 4.07/62.28 | 0.34/0.04 |
| 1SS model+DTT | 4.11/62.56 | 0.07/0 | 4.22/53.00 | 0.22/0 | 4.08/62.24 | 0.38/0 |
| random coil[d] | 4.16/62.61 | / | 4.35/52.82 | / | 4.16/62.61 | / |

[a]All chemical shifts were calibrated in parts per million (ppm) relative to 4,4-dimethyl-4-silapentane-1-sulfonic acid (DSS) as an internal standard, which was set to 0 ppm.

[b]The $^1H_\alpha/^{13}C_\alpha$ secondary chemical shift was calculated according the equation $\Delta_{H\alpha/C\alpha} = \sqrt{\delta_{H\alpha}^2 + \delta_{C\alpha}^2}$. $\delta_{H\alpha}$ and $\delta_{C\alpha}$ are the $^1H_\alpha$ and $^{13}C_\alpha$ secondary chemical shift and are the differences between observed chemical shift of DesDi variants and random coil shifts from literature (Δ1), or the shifts of random coil base line of single-chain DesDi 1SS model in 70 mM deuterated dithiothreitol (DTT) (Δ2).

[c]The $^{13}C_\alpha$ resonance of Ala[B14] shifted toward upfield due to proline replacement at B15 position; The secondary shift of alanine in 1SS-Pro[B15] was calculated using Ala[A11].

[d]The $^1H_\alpha/^{13}C_\alpha$ chemical shifts of random coil were measured in aqueous solutions of the linear pentapeptide (GGXGG) (29).

**TABLE S7**. The $^1H_\alpha$ chemical shift dispersion of different DesDi analogs at 35 °C[a]

| | $^1H\alpha$ range (ppm) | $^1H\alpha$ dispersion (ppm) | relative $^1H\alpha$ dispersion (ppm) |
|---|---|---|---|
| single-chain N* | 3.26-5.40 | 2.14 | 1.33 |
| two-chain N* | 3.20-5.30 | 2.10 | 1.29 |
| 1SS model | 3.32-5.13 | 1.81 | 1.00 |
| 1SS-Ser[B24] | 3.68-4.83 | 1.15 | 0.34 |
| 1SS-Pro[A16] | 3.77-4.81 | 1.04 | 0.23 |
| 1SS-Pro[B15] | 3.82-4.71 | 0.89 | 0.08 |
| 1SS model+DTT | 3.84-4.65 | 0.81 | / |

[a]All chemical shifts were calibrated in parts per million (ppm) relative to 4,4-dimethyl-4-silapentane-1-sulfonic acid (DSS) as an internal standard, which was set to 0 ppm.

[b]Relative $^1H\alpha$ chemical shift dispersion was defined as the difference between observed $^1H\alpha$ chemical shift dispersion and the random coil base line which is the $^1H\alpha$ chemical shift dispersion observed for the single-chain DesDi 1SS model in 70 mM deuterated dithiothreitol (DTT).

**TABLE S8**. The $^1H_\alpha$ chemical shift dispersion of different DesDi analogs at 10 °C[a]

| | $^1H\alpha$ range (ppm) | $^1H\alpha$ dispersion (ppm) | relative $^1H\alpha$ dispersion[b] (ppm) |
|---|---|---|---|
| single-chain N* | / | / | / |
| two-chain N* | 3.21-5.32 | 2.11 | 1.29 |
| 1SS model | 3.21-5.25 | 2.04 | 1.22 |
| 1SS-Ser$^{B24}$ | 3.61-4.96 | 1.35 | 0.53 |
| 1SS-Pro$^{A16}$ | 3.66-4.93 | 1.27 | 0.45 |
| 1SS-Pro$^{B15}$ | 3.79-4.75 | 0.96 | 0.14 |
| 1SS model+DTT | 3.83-4.65 | 0.82 | / |

[a]All chemical shifts were calibrated in parts per million (ppm) relative to 4,4-dimethyl-4-silapentane-1-sulfonic acid (DSS) as an internal standard, which was set to 0 ppm.

[b]Relative $^1H\alpha$ chemical shift dispersion was defined as the difference between observed $^1H\alpha$ chemical shift dispersion and the random coil base line which is the $^1H\alpha$ chemical shift dispersion observed for the parent 1SS model in 70 mM deuterated dithiothreitol (DTT).

**TABLE S9.** Secondary chemical shift of amide-proton, alpha-proton, alpha-carbon and beta-carbon in 1SS-DesDi model at 35°C[a,b,c]

| B domain | | | | | | A domain | | | | |
|---|---|---|---|---|---|---|---|---|---|---|
| residual | $\Delta H_N$ | $\Delta H_\alpha$ | $\Delta C_\alpha$ | $\Delta C_\beta$ | | residual | $\Delta H_N$ | $\Delta H_\alpha$ | $\Delta C_\alpha$ | $\Delta C_\beta$ |
| F1 | | -0.48 | -0.69 | 1.82 | | G29 | -0.28 | 0.48 | 0.22 | |
| V2 | -0.10 | -0.29 | 1.56 | 0.25 | | I30 | -0.34 | -0.06 | 0.57 | 0.46 |
| N3 | -0.01 | -0.11 | 0.55 | 0.98 | | V31 | -0.22 | -0.04 | -0.14 | 0.51 |
| Q4 | 0.13 | 0.04 | 0.45 | 2.68 | | E32 | -0.25 | -0.01 | -0.49 | -0.21 |
| H5 | 0.04 | 0.03 | 1.37 | 2.63 | | Q33 | -0.01 | -0.05 | -0.09 | 1.46 |
| L6 | 0.01 | 0.15 | -0.52 | 1.61 | | A34 | -0.17 | 0.02 | 0.64 | 0.45 |
| S7 | 0.14 | 0.19 | -0.74 | 0.90 | | S35 | -0.24 | 0.16 | -0.08 | 0.60 |
| G8 | 0.31 | -0.7 | 1.67 | | | E36 | | | | -0.27 |
| S9 | | -0.21 | 2.85 | -0.17 | | S37 | | 0.01 | -0.44 | 1.86 |
| D10 | -0.29 | -0.17 | 2.33 | 0.12 | | I38 | -0.01 | 0.08 | -0.81 | 1.01 |
| L11 | -0.74 | -0.39 | 2.10 | -0.96 | | A39 | 0.29 | 0.02 | 0.63 | 0.78 |
| V12 | -0.84 | -0.80 | 3.95 | -0.47 | | S40 | -0.53 | 0.07 | 0.38 | 0.79 |
| E13 | -0.46 | -0.27 | 2.69 | -0.03 | | L41 | 0.28 | -0.30 | 1.83 | -0.50 |
| A14 | -0.51 | -0.20 | 2.27 | -0.31 | | Y42 | 0.21 | -0.15 | 1.05 | -0.83 |
| L15 | -0.21 | -0.55 | 2.33 | -1.37 | | Q43 | -0.49 | -0.10 | 1.27 | 0.10 |
| Y16 | -0.27 | -0.13 | 2.92 | -0.77 | | L44 | -0.35 | -0.13 | 1.64 | -0.34 |
| L17 | -0.54 | -0.23 | 2.86 | 0.03 | | E45 | -0.34 | -0.10 | 1.85 | -0.14 |
| V18 | -0.13 | -0.26 | 2.19 | 0.85 | | N46 | -0.92 | -0.26 | 1.98 | 0.35 |
| C19 | 0.32 | 0.10 | -1.09 | | | Y47 | -0.41 | 0.04 | 0.29 | -0.13 |
| G20 | -0.41 | 0.02 | 1.41 | | | C48 | -0.69 | 0.33 | -1.68 | |
| E21 | 0.60 | -0.17 | 1.10 | -0.16 | | N49 | -0.40 | -0.24 | 2.34 | 1.47 |
| R22 | -0.19 | -0.14 | 1.74 | 0.75 | | | | | | |
| G23 | -0.88 | 0.13 | -0.31 | | | | | | | |
| F24 | -0.53 | 0.51 | -1.65 | 3.34 | | | | | | |
| F25 | 0.42 | 0.16 | -0.77 | 1.26 | | | | | | |
| Y26 | 0.07 | -0.11 | 0.31 | 0.66 | | | | | | |
| T27 | | -0.18 | -0.49 | 0.57 | | | | | | |
| K28 | | -0.10 | 0.64 | 0.19 | | | | | | |

[a]All chemical shifts were calibrated in parts per million (ppm) relative to 4,4-dimethyl-4-silapentane-1-sulfonic acid (DSS) as an internal standard, which was set to 0 ppm.

[b]All of the secondary chemical shift was calculated according the equation ($\Delta = \delta_{obs} - \delta_{coil}$). $\delta_{obs}$ are observed $^1H_N$, $^1H_\alpha$, $^{13}C_\alpha$ and $^{13}C_\beta$ chemical shift; $\delta_{coil}$ are the chemical shift of random coil for $^1H_N$, $^1H_\alpha$, $^{13}C_\alpha$ and $^{13}C_\beta$ (46, 47).

[c]The secondary chemical shift of amide-proton was extracted from 1SS-Glu$^{A14}$ DesDi variant.

## References


1. Anfinsen CB. Principles that govern the folding of protein chains. *Science*. 1973;181(4096):223-30.
2. Baldwin RL. The nature of protein folding pathways: the classical versus the new view. *J Biomol NMR*. 1995;5(2):103-9.
3. Sali A, Shakhnovich E, Karplus M. How does a protein fold? *Nature*. 1994;369(6477):248-51.
4. Creighton TE. Characterizing intermediates in protein folding. *Curr Biol*. 1991;1(1):8-10.
5. Goldenberg DP. Kinetic analysis of the folding and unfolding of a mutant form of bovine pancreatic trypsin inhibitor lacking the cysteine-14 and-38 thiols. *Biochemistry*. 1988;27(7):2481-9.
6. Staley JP, Kim PS. Role of a subdomain in the folding of bovine pancreatic trypsin inhibitor. *Nature*. 1990;344(6267):685-8.
7. Weissman JS, Kim PS. Kinetic role of nonnative species in the folding of bovine pancreatic trypsin inhibitor. *Proc Natl Acad Sci USA*. 1992;89(20):9900-4.
8. Staley JP, Kim PS. Complete folding of bovine pancreatic trypsin inhibitor with only a single disulfide bond. *Proc Natl Acad Sci USA*. 1992;89(5):1519-23.
9. Dadlez M, Kim PS. A third native one-disulphide intermediate in the folding of bovine pancreatic trypsin inhibitor. *Nat Struct Biol*. 1995;2(8):674-9.
10. Kibria FM, Lees WJ. Balancing conformational and oxidative kinetic traps during the folding of bovine pancreatic trypsin inhibitor (BPTI) with glutathione and glutathione disulfide. *J Am Chem Soc*. 2008;130(3):796-7.
11. Kurokawa Y, Koganesawa N, Kobashigawa Y, Koshiba T, Demura M, Nitta K. Oxidative folding of human lysozyme: effects of the loss of two disulfide bonds and the introduction of a calcium-binding site. *J Protein Chem*. 2001;20(4):293-303.
12. Gurbhele-Tupkar MC, Perez LR, Silva Y, Lees WJ. Rate enhancement of the oxidative folding of lysozyme by the use of aromatic thiol containing redox buffers. *Biorg Med Chem*. 2008;16(5):2579-90.
13. Madar DJ, Patel AS, Lees WJ. Comparison of the oxidative folding of lysozyme at a high protein concentration using aromatic thiols versus glutathione. *J Biotechnol*. 2009;142(3-4):214-9.
14. Arai K, Shibagaki W, Shinozaki R, Iwaoka M. Reinvestigation of the oxidative folding pathways of hen egg white lysozyme: Switching of the major pathways by temperature control. *Int J Mol Sci*. 2013;14(7):13194-212.
15. Narayan M, Welker E, Wedemeyer WJ, Scheraga HA. Oxidative folding of proteins. *Acc Chem Res*. 2000;33(11):805-12.
16. Wedemeyer WJ, Welker E, Narayan M, Scheraga HA. Disulfide bonds and protein folding. *Biochemistry*. 2000;39(15):4207-16.
17. Welker E, Narayan M, Wedemeyer WJ, Scheraga HA. Structural determinants of oxidative folding in proteins. *Proc Natl Acad Sci USA*. 2001;98(5):2312-6.
18. Welker E, Wedemeyer WJ, Narayan M, Scheraga HA. Coupling of conformational folding and disulfide-bond reactions in oxidative folding of proteins. *Biochemistry*. 2001;40(31):9059-64.
19. Hua QX, Mayer J, Jia W, Zhang J, Weiss MA. The folding nucleus of the insulin superfamily: a flexible peptide model foreshadows the native state. *J Biol Chem*. 2006;281:28131-42.
20. Yan H, Guo ZY, Gong XW, Xi D, Feng YM. A peptide model of insulin folding intermediate with one disulfide. *Protein Sci*. 2003;12:768-75.
21. Hua QX, Narhi L, Jia W, Arakawa T, Rosenfeld R, Hawkins N, et al. Native and non-native structure in a protein-folding intermediate: spectroscopic studies of partially reduced IGF-I and an engineered alanine model. *J Mol Biol*. 1996;259:297-313.
22. Miller JA, Narhi LO, Hua QX, Rosenfeld R, Arakawa T, Rohde M, et al. Oxidative refolding of insulin-like growth factor 1 yields two products of similar thermodynamic stability: a bifurcating protein-folding pathway. *Biochemistry*. 1993;32:5203-13.
23. Jia XY, Guo ZY, Wang Y, Xu Y, Duan SS, Feng YM. Peptide models of four possible insulin folding intermediates with two disulfides. *Protein Sci*. 2003;12:2412-9.

24. Dhayalan B, Glidden MD, Zaykov A, Chen Y-S, Yang Y, Phillips NB, et al. Peptide Model of the Mutant Proinsulin Syndrome. I. Design and Clinical Correlation. 2022:companion paper (submitted).
25. Rege NK, Liu M, Yang Y, Dhayalan B, Wickramasinghe NP, Chen Y-S, et al. Evolution of insulin at the edge of foldability and its medical implications. *Proc Natl Acad Sci USA*. 2020;117(47):29618-28.
26. Barbar E, Barany G, Woodward C. Dynamic Structure of a Highly Ordered. β-Sheet Molten Globule: Multiple Conformations with a Stable Core. *Biochemistry*. 1995;34(36):11423-34.
27. Li R, Woodward C. The hydrogen exchange core and protein folding. *Protein Sci*. 1999;8(8):1571-90.
28. Qiao Z-S, Min C-Y, Hua Q-X, Weiss MA, Feng Y-M. In vitro refolding of human proinsulin kinetic intermediates, putative disulfide-forming pathway, folding initiation site, and potential role of C-peptide in folding process. *J Biol Chem*. 2003;278(20):17800-9.
29. Schwarzinger S, Kroon GJ, Foss TR, Wright PE, Dyson HJ. Random coil chemical shifts in acidic 8 M urea: implementation of random coil shift data in NMRView. *J Biomol NMR*. 2000;18(1):43-8.
30. Dyson HJ, Wright PE. Unfolded proteins and protein folding studied by NMR. *Chem Rev*. 2004;104(8):3607-22.
31. Dyson HJ, Ewright P. Insights into the structure and dynamics of unfolded proteins from nuclear magnetic resonance. *Adv Protein Chem*. 2002;62:311-40.
32. Yao J, Dyson HJ, Wright PE. Chemical shift dispersion and secondary structure prediction in unfolded and partly folded proteins. *FEBS Lett*. 1997;419(2-3):285-9.
33. Goldenberg DP. Principles of NMR spectroscopy: an illustrated guide: University Science Books; 2016.
34. Korzhnev DM, Kay LE. Probing invisible, low-populated states of protein molecules by relaxation dispersion NMR spectroscopy: an application to protein folding. *Acc Chem Res*. 2008;41(3):442-51.
35. Hill RB, Bracken C, DeGrado WF, Palmer AG. Molecular motions and protein folding: characterization of the backbone dynamics and folding equilibrium of $\alpha_2$D using $^{13}$C NMR spin relaxation. *J Am Chem Soc*. 2000;122(47):11610-9.
36. Sandström J, Sandström JS. Dynamic NMR spectroscopy: Academic Press; 1982.
37. Steigel A, Spiess HW. Dynamic NMR spectroscopy: Springer Science & Business Media; 2012.
38. Barbar E, Barany G, Woodward C. Unfolded BPTI variants with a single disulfide bond have diminished non-native structure distant from the crosslink. *Folding and Design*. 1996;1(1):65-76.
39. van Mierlo CP, Darby NJ, Neuhaus D, Creighton TE. Two-dimensional $^1$H nuclear magnetic resonance study of the (5–55) single-disulphide folding intermediate of bovine pancreatic trypsin inhibitor. *J Mol Biol*. 1991;222(2):373-90.
40. Ferrer M, Barany G, Woodward C. Partially folded, molten globule and molten coil states of bovine pancreatic trypsin inhibitor. *Nat Struct Biol*. 1995;2(3):211-7.
41. van Mierlo CP, Darby NJ, Keeler J, Neuhaus D, Creighton TE. Partially folded conformation of the (30-51) intermediate in the disulphide folding pathway of bovine pancreatic trypsin inhibitor: $^1$H and $^{15}$N resonance assignments and determination of backbone dynamics from $^{15}$N relaxation measurements. *J Mol Biol*. 1993;229(4):1125-46.
42. Sreerama N, Woody RW. A self-consistent method for the analysis of protein secondary structure from circular dichroism. *Anal Biochem*. 1993;209(1):32-44.
43. Sreerama N, Woody RW. Poly (Pro) II helixes in globular proteins: Identification and circular dichroic analysis. *Biochemistry*. 1994;33(33):10022-5.
44. Johnson WC. Analyzing protein circular dichroism spectra for accurate secondary structures. *Proteins: Struct Funct Bioinform*. 1999;35(3):307-12.
45. Hua Q-X, Weiss MA. Mechanism of insulin fibrillation: the structure of insulin under amyloidogenic conditions resembles a protein-folding intermediate. *J Biol Chem*. 2004;279(20):21449-60.
46. Wishart DS, Sykes BD, Richards FM. The chemical shift index: a fast and simple method for the assignment of protein secondary structure through NMR spectroscopy. *Biochemistry*. 1992;31(6):1647-51.
47. Wishart DS, Sykes BD. The $^{13}$C chemical-shift index: a simple method for the identification of protein secondary structure using $^{13}$C chemical-shift data. *J Biomol NMR*. 1994;4(2):171-80.
